# The Role of Spatially Controlled Cell Proliferation in Limb Bud Morphogenesis

**DOI:** 10.1371/journal.pbio.1000420

**Published:** 2010-07-13

**Authors:** Bernd Boehm, Henrik Westerberg, Gaja Lesnicar-Pucko, Sahdia Raja, Michael Rautschka, James Cotterell, Jim Swoger, James Sharpe

**Affiliations:** 1 EMBL-CRG Systems Biology Research Unit, Centre for Genomic Regulation (CRG), UPF, Barcelona, Spain; 2 MRC Human Genetics Unit, Edinburgh, Scotland, United Kingdom; 3 ICREA Professor, Centre for Genomic Regulation (CRG), UPF, Barcelona, Spain; University of Cambridge, United Kingdom

## Abstract

Although the vertebrate limb bud has been studied for decades as a model system for spatial pattern formation and cell specification, the cellular basis of its distally oriented elongation has been a relatively neglected topic by comparison. The conventional view is that a gradient of isotropic proliferation exists along the limb, with high proliferation rates at the distal tip and lower rates towards the body, and that this gradient is the driving force behind outgrowth. Here we test this hypothesis by combining quantitative empirical data sets with computer modelling to assess the potential role of spatially controlled proliferation rates in the process of directional limb bud outgrowth. In particular, we generate two new empirical data sets for the mouse hind limb—a numerical description of shape change and a quantitative 3D map of cell cycle times—and combine these with a new 3D finite element model of tissue growth. By developing a parameter optimization approach (which explores spatial patterns of tissue growth) our computer simulations reveal that the observed distribution of proliferation rates plays no significant role in controlling the distally extending limb shape, and suggests that directional cell activities are likely to be the driving force behind limb bud outgrowth. This theoretical prediction prompted us to search for evidence of directional cell orientations in the limb bud mesenchyme, and we thus discovered a striking highly branched and extended cell shape composed of dynamically extending and retracting filopodia, a distally oriented bias in Golgi position, and also a bias in the orientation of cell division. We therefore provide both theoretical and empirical evidence that limb bud elongation is achieved by directional cell activities, rather than a PD gradient of proliferation rates.

## Abstract 2

Oriented cell behaviors likely have a more important role in limb bud elongation during development than previously suggested by the “growth-based morphogenesis” hypothesis.

## Author Summary

Although the vertebrate limb bud has been studied for decades as a classical model system for the spatial control of cell fates, the question of how the limb bud physically elongates has been much less studied. One particular hypothesis has been dominant in the field, known either as the *proliferation gradient hypothesis* or *growth-based morphogenesis*. This states that elongation is achieved by distal cells (furthest from the body) being stimulated to divide faster than proximal cells. Importantly, this hypothesis does not propose any kind of oriented or directional cell behaviours—high distal rates of *non-oriented* proliferation are considered to be sufficient—and indeed several 2D computer simulations have reproduced this concept *in silico*. However, thus far quantitative data from the limb bud has not been incorporated into these models. Here, we extended computer simulations into 3D and incorporated quantitative data on both shape changes and proliferation rates. These new simulations demonstrated that gradients of non-oriented proliferation are unable to explain limb bud elongation. We thus experimentally tested for evidence of oriented cell behaviours and indeed found that the cell shape, Golgi orientation, and cell divisions all display a non-random bias during limb bud outgrowth. Our data run contrary to the proliferation gradient hypothesis, indicating instead that oriented cell behaviours are important for driving elongation.

## Introduction

**Figure 1:**
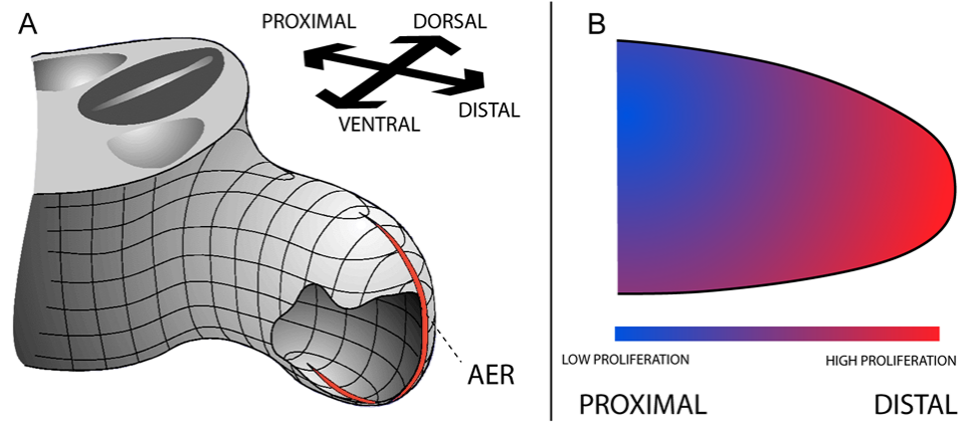
Gradient based morphogenesis. (A) Schematic of the 3D organization of the vertebrate limb bud. Elongation occurs along the proximo-distal axis (away from the body), and the apical ectodermal ridge (AER) runs along the distal-most part of the bud. (B) The proliferation gradient model (or *growth-based morphogenesis*, Morishita and Iwasa [23]) proposes that a zone of high proliferation close to the AER drives distally directed limb elongation.

Vertebrate limb development is a classical model system which has contributed to some of the key concepts in the developmental field [1],[2]. Over a period of 1–2 d a roughly homogeneous mass of undifferentiated mesenchymal cells develops into a complex collection of different cell types (including cartilage, bone, tendons, and dermis) which are spatially organized to create a functional organ. In the mouse and chick, the limb bud starts as a small bulge from the lateral flank of the embryo. It possesses a very simple structure, composed of a central mass of mesenchymal cells covered by a single-cell ectodermal epithelium. Very early in development the ectoderm develops a thickened ridge (the apical ectodermal ridge, or AER) which runs along the antero-posterior (AP) axis and marks the anatomical boundary between dorsal and ventral ectoderm (Figure 1A). Subsequent growth of the bud shows a preferential orientation—extension in the distal direction (away from the body) is dramatic, while by comparison the increase in width and height is much slower. Although great strides have been made in understanding the molecular basis of patterning and cell specification [3],[4],[5],[6],[7], the cellular basis of distally oriented limb bud outgrowth remains unclear.

The oldest and still the most prominent hypothesis to explain the physical morphogenesis of limb bud outgrowth is the “proliferation gradient” model (Figure 1B), first described by Ede and Law in 1969 [8]. This idea states that a diffusible signal from the AER acts primarily as a mitogen [9] which “…signals the mesenchyme immediately underlying it, termed the progress or proliferative zone, to proliferate, resulting in directed proximo-distal outgrowth” [10]. Much evidence appears to support this hypothesis, in particular a graded distribution of proliferation rates along the proximo-distal axis has been reported for both mouse and chick limb buds (as observed by mitotic index counting [11], BrdU incorporation [12], [^3^H]thymidine labelling [9], and cell-cycle specific antibody labelling [13]). During the 1990s concrete evidence was presented for the molecules responsible for this mitogenic effect—FGF4 and FGF8 were shown to be expressed specifically in the AER, to be able to diffuse away from the source cells [14] and to have mitogenic influence in cell culture and various organ systems [10],[15],[16].

A few other hypotheses have been proposed to explain limb bud morphogenesis. Oriented cell divisions are known to be involved in other morphogenetic processes like vertebrate gastrulation [17] or *Drosophila* wing disc development [18], however no evidence for a preferred direction of cell division has been shown during limb bud extension [11] or subsequently been reported. Programmed cell death is known to occur in a few localized regions of the developing bud [13], but it cannot explain the distally directed extension of the limb. More importantly, Li and Muneoka [19] performed an elegant experiment demonstrating that at least some of the mesenchymal cells in the chick wing bud can move towards an ectopic source of FGF4 implanted in the centre of the bud. This raised the intriguing possibility that mesenchymal cells might treat the FGF gradient as a chemoattractant rather than a mitogen, and respond by active migration towards the AER. Finally, it was speculated in 1970 [11] that the ectoderm might play a mechanical role in shaping the growing limb bud, however it has been demonstrated that limb development can proceed quite normally in the absence of significant regions of dorsal ectoderm [20],[21].

Despite these suggested alternatives, the “proliferation gradient” model has remained the dominant hypothesis for the last 40 years [22],[23],[24]. Although the concept was first articulated by Ede and Law [8], it was their own pioneering computer simulation (from the late 1960s) which first questioned the model. However, the strength of their conclusions rested on a rather abstract 2D cellular automaton approach, and more recent computer simulations have adopted the idea. In 1999 Dillon and Othmer [22] created the first realistic 2D finite element model (FEM) of limb development, and although their simulation was not aimed at exploring the cellular basis of directional outgrowth, they nevertheless directly incorporated the proliferation gradient hypothesis into their model by allowing growth rates to be controlled by a molecule diffusing from the distal tip. In contrast to Dillon and Othmer, more recent computer simulations have specifically aimed to explore the proliferation gradient model and concluded that the hypothesis can indeed explain limb outgrowth. Poplawski et al. incorporated the gradient hypothesis within a Cellular Potts framework to show that distally restricted growth could produce bud elongation [25], while Morishita and Iwasa employed a cell-based 2D spring lattice [23] to demonstrate a similar result. This last study renames the concept with the more explicit term “*growth-based morphogenesis*,” emphasizing that although the global shape changes are directional, they result from local cell behaviours which are non-directional [23]. This highlights our key question to be addressed here: Are the important cell activities for outgrowth *directional* or *isotropic*?

All active cell behaviours can be classified into these two categories. Processes such as migration and intercalation are directional—they depend on cells having a sense of orientation (for example by sensing gradients or using the PCP system). By contrast, behaviours such as cell death are not oriented (i.e. they are *isotropic*), and therefore cause contraction of tissue equally in all directions. Cell proliferation can fall into either of these two categories: in some cases the orientation of cell division is known to be carefully controlled (like *Drosophila* wing disc development [18]), but in other cases there appears to be no control of orientation, for example in the developing limb bud it is believed that cells divide randomly in any orientation [11]. The popular notion of *growth-based morphogenesis* (which is equivalent to the established proliferation gradient model) states that controlling the *rates* of isotropic proliferation is enough [23], and that cells do not require directional information.

Here we develop a new interdisciplinary approach to address this question. Rather than explore a range of different hypotheses, our goal is to focus on the most popular one—*growth-based morphogenesis* [23]—and to rigorously test its sufficiency as an explanation of limb bud outgrowth. In other words, we explore whether non-directional (isotropic) cell behaviours can explain limb bud elongation. Firstly, we develop novel data-capture and processing techniques to generate empirical quantitative data-sets for two distinct aspects of the developing mouse limb bud: (i) accurate 3D geometry of the shape changes and (ii) a quantitative map of cell cycle times. Secondly, to our knowledge, we create the first dynamic 3D computer simulation of limb outgrowth, which is directly based on these data. By developing a novel 3D parameter-optimisation approach we demonstrate that the observed spatial control of proliferation rates has little impact on morphogenesis and cannot explain distally directed limb bud outgrowth. We conclude that isotropic cell behaviours in general, such as non-oriented cell proliferation and programmed cell death, provide little or no contribution to the major observed shape changes. The main role of proliferation must be simply to provide enough progenitor cells for the organ. We predict that shape generation (elongation) must instead be driven by directional cell activities, such as active migration or cell intercalation. Prompted by this theoretical prediction, we go back to the real system to examine cell shape and markers of cell orientation in the developing chick limb bud. We find that most mesenchymal cells have a striking highly branched and extended cell shape composed of dynamically extending and retracting filopodia, a distally oriented bias in Golgi position, and also a bias in the orientation of cell division. The discovery of this collection of oriented cellular activities opens a new area of enquiry into how active mesenchymal behaviours (such as migration and intercalation) contribute to morphogenesis of the limb, which has only been addressed by one pioneering study so far [19].

## Results

### OPT Can Accurately Measure the 3D Geometry of Limb Bud Shapes

**Figure 2:**
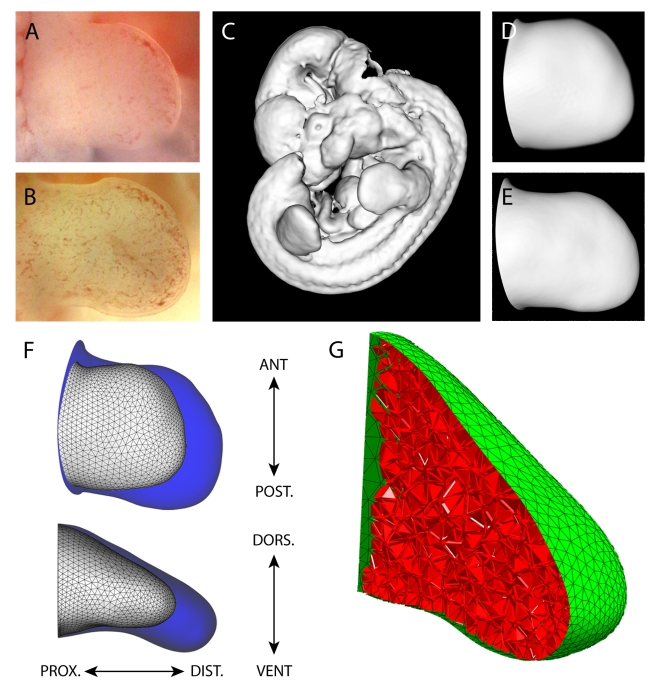
The changing 3D geometry during limb development. (A) The right hind limb bud at stage E11.0 from the freshly dissected embryo, and (B) a right hind limb bud from another embryo at stage E11.25, 6 h older than (A). (C) The OPT scans were converted into an iso-surface, a 3D contour of the embryo. The limb bud shapes *S_t0_*, (D) and *S_t1_* (E) were virtually dissected from the whole-embryo iso-surface. (F) A comparison between the two empirically measured shapes *S_t0_* (white) and *S_t1_* (blue) which highlights the shape change over 6 h of development. The main axes are shown anterio-posterior (AP), dorso-ventral (DV), and proximo-distal (PD). (G) A virtual slice through the fully tetrahedtralised *S_t0_* mesh. The surface is discretised with a triangular mesh (green), and the internal mesenchymal volume is discretised with a 3D tetrahedral mesh (red).

To build a data-driven computer model we wished to have an accurate representation of the changing shape of the growing mouse limb bud. A numerical simulation requires a defined spatial domain, and previous studies have employed 2D abstract approximations of the growing limb bud shapes. In this study we have improved on previous work in two ways: Firstly, the simulation (and therefore the shape information) is 3D instead of 2D. This is important as we aim to model mechanical forces which cannot be correctly captured in a 2D abstraction (for example the 2D simulation of Dillon and Othmer [22] required virtual springs to be added from the dorsal ectoderm to the ventral ectoderm, to compensate for the lack of the full 3D structure). Secondly, rather than a simple mathematically defined “bulging” shape, we wished to use real empirical shape features to define the spatial domain of our simulation. We therefore employed state-of-the-art 3D imaging technology to generate accurate quantitative shape information of real embryonic mouse limb buds (Figure 2).

For each embryo examined, detailed 3D shape information was generated using optical projection tomography (OPT)—an imaging technology ideally suited for scanning mesoscopic biological specimens [26]. Using an OPT scanner we captured 400 projection images of the autofluorescence from the embryo, rotating it by 0.9° between images. A filtered back-projection algorithm [27] was then used to reconstruct a voxel dataset from the raw projections, and from this a 3D intensity iso-surface was generated for the embryo (this is a 3D contour which encloses all regions above a certain threshold intensity, Figure 2C). From these static 3D representations, the right hind limb was then virtually dissected away from the rest of the embryo for further analysis (Figure 2D,2E).

We selected two limb buds of different developmental ages for 3D scanning: an earlier stage with shape denoted by *S_t0_* representing the initial condition for the simulation and a later stage *S_t1_* which defines the target shape that real limb development achieves. These shapes are given by the isosurfaces extracted from the empirical OPT data, as described in the previous paragraph. The general strategy followed is to simulate the growth of the limb starting with initial shape *S_t0_* and then to compare the shapes predicted by the simulation (predicted = *pS_t1_*) with the real target shape *S_t1_*. Three criteria were important in choosing suitable developmental stages for *S_t0_* and *S_t1_*. The shape change must be significant enough to distinguish between good and bad results of the simulation, but the time-interval should also be short enough to be computationally efficient. Also, for the purposes of building the computer model we wish to assume uniform physical properties for the mesenchyme, and we therefore require a developmental stage when internal mesenchymal condensations have not yet formed. Based on these criteria we selected two limb buds (E11.0 and E11.25) which are 6 h apart in normal development (see Figure 2A,B). The shapes of six embryos at each stage were examined to ensure that the shape change due to growth was much greater than the shape variability between embryos of the same stage (Figure S1). Mesenchymal cell density was also checked at different positions of the older limb bud (E11.25), and despite the expression of early skeletal markers such as Sox9 and Noggin, localised regions of higher density (mesenchymal condensations) have not yet formed (Figure S2). Staging was performed by comparison to a collection of 200 embryos harvested at precise time-points between E10.5 and E12.5. The resulting isosurface *S_t0_* could then be used to create the full 3D tetrahedral mesh required for subsequent modelling (Figure 2G; this was done using the program NetGen [28]).

We have therefore created for the first time a numerical (geometric) representation of the mouse limb bud shape change in 3D. Using this resource we can assess the limb bud length (PD) of *S_t0_* and *S_t1_* (1,020 µm and 1,450 µm, respectively) and calculate an average extension rate of 75 microns/h, or a total extension of 43% over 6 h (note that the scales in Figures 2A–E are not the same). In contrast, the extension along the DV and AP axis is much less: ∼1% and 12%, respectively. The volumes for the two time-points are 0.551 mm^3^ and 0.914 mm^3^, respectively.

### Measuring and Mapping the 3D Distribution of Cell Proliferation Rates

The second dataset required for our study is a quantitative 3D map of cell proliferation rates in the limb. Common approaches for measuring cell proliferation include mitotic index counting, BrdU, and anti-pH3 staining. In 1970 Hornbruch and Wolpert [11] used haematoxylin and eosin staining to analyze the mitotic index of mesenchymal cells in the chick limb and identified a gradient of high proliferation at the distal tip and lower proliferation proximally at stages HH23–27. More recently Fernández-Terán et al. [13] studied the cell proliferation activity in the mouse and chick limbs using anti-pH3 immunohistochemistry and found a similar general pattern. However, single labelling techniques like these have an important limitation. Determining the number of cells in one cell cycle phase as a fraction of the total number of cells (for example the proportion of BrdU-labelled cells in S-phase) is a relative measurement and cannot provide information on how long that phase lasts in minutes or hours. This limitation applies to all single-labelling approaches, whether using BrdU, pH3, Ki67, PCNA, or tritiated-thymidine. Previous studies have therefore provided information on the relative rates across different regions of the limb, but have never quantified these spatial patterns in terms of cell cycle time.

**Figure 3:**
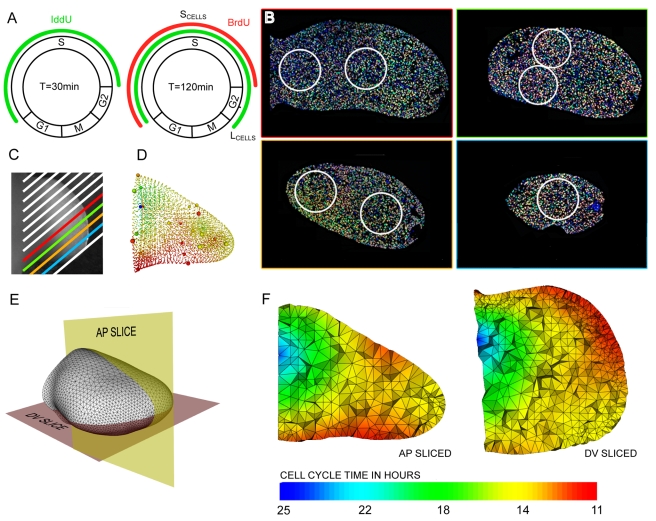
A quantitative 3D map of proliferation rates. (A) The four phases of the cell cycle: Gap 1 (G1), Synthesis (S-phase), Gap 2 (G2), and Mitosis (M phase). IddU was injected into the mouse and incorporated into the limb bud cells in S-phase (left-hand side, green bar). After time interval *Ti* a second injection is made, this time of BrdU, which again labels the cells in S-phase at that moment (right-hand side, red bar). At this point some of the IddU-labelled cells from the first injection (green) have left S-phase and are now in G2. These are the “Leaving cells,” or L_cells_, and were counted to calculate *Tc* using equation (2). (B) 7 µm thick paraffin sections were imaged by confocal microscopy. For each of the 30 counting regions (white circles on sections, 215 µm diametre), the total number of cells is counted and also the number of L_cells_ (blue and green IddU-labelled cells, which display no red). (C) The positions of the 13 sections overlaid onto a photo of the same limb. (D) The calculated *T_c_* values are assigned to the corresponding vertices on the 3D mesh (indicated by small coloured spheres) and are interpolated onto the remaining vertices. (E) Schematic of virtual sections anterio-posterior (AP) and dorso-ventral (DV). (F) Colors show the distribution of the *T_c_* values in hours for the virtual sections indicated in (E). A core of low proliferation (proximal central) and areas of high proliferation (dorsal and ventral), as well as a ridge of high proliferation (anterior-distal) can be seen.

Pulse-chase experiments overcome this limitation through the use of two or more labels administered to the living cells at different times [29]; however, this has typically been done on dissociated cell populations, thereby losing all spatial information. To overcome this problem we adapted a double-labelling technique successfully used by Martynoga et al. [30] to quantify proliferation rates on 2D sections of the developing telencephalon (adapted from Shibui et al. [31]). This approach allows measurement of the average cell cycle time of a population of cells by sequentially labelling them with two different markers at a known time interval (Figure 3A). Pregnant females are injected first with IddU and then after time interval T_i_ are injected again with BrdU. Embryos harvested 30 min later are fixed, embedded, sectioned, and then analysed using two different fluorescent secondary antibodies plus DAPI staining, which allows the identification of three cell populations: unlabelled (blue), single-labelled (blue and green), and double-labelled. In effect this creates a new artificial phase of the cell cycle whose exact duration is known: single-labelled cells are those which left S-phase during time T_i_ (called *leaving cells*, the number of which is L_cells_ [29]). Since the cell population is dividing asynchronously, then the different phases of the cell cycle will be sampled equally (Figure 3A). The ratio of the total number of cells to L_cells_ therefore equals the ratio of the total cell cycle, T_c_, to T_i_:
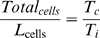
(1)T_c_ can therefore easily be calculated for each local population of labelled cells:
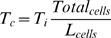
(2)To create our quantitative 3D map of cell proliferation rates a limb bud was chosen (having an age in between those of *S_t0_* and *S_t1_*) and was cut into sections 7 µm thick (Figure 3B,C). After fluorescent immunohistochemistry we analysed how smoothly the T_c_ values vary across the sections and subsequently selected 30 areas to capture this spatial distribution. For each of these areas the differently labelled cells in a circular region with a diameter of 215 µm were manually counted and the cell-cycle time calculated (Figure 3B). A 3D mapping between the 30 areas and *S_t0_* was created (Figure 3D). This gave a sparse representation of proliferation rates for the limb. These values were then interpolated across the remaining vertices of the tetrahedral mesh corresponding to *S_t0_* (from Figure 2G) using a radial basis function (RBF, see Materials and Methods), creating a full map of T_c_ values which vary smoothly across the 3D space of the limb bud.

We have thus created the first quantitative 3D map of proliferation rates for a growing vertebrate limb bud. The qualitative pattern agrees with previous studies (with higher proliferation rates in distal regions and closer to the ridge, and lower rates at the proximal end) [13], however a map of absolute T_c_ values across the tissue has not previously been achieved. We performed this analysis for a few limb buds at different ages and revealed that over the 6 h period from E11.0 to E11.25 the changes in proliferation rate are insignificant. This makes sense when one considers that the fastest cell cycle time was itself ∼10 h. We therefore consider this 3D map to be a suitable representation of the average rates during the 6 h period of the simulation.

### A 3D FEM of Limb Bud Growth

The next aspect of this study was to define a suitable method for integrating the data into a dynamic model of growth. Three components are required: a method to represent the growing 3D spatial domain of the simulation, a choice of model/equations to represent the growing limb bud tissue and a numerical method to solve the equations over time.

Previous models (which have all been 2D) have included a variety of approaches, either considering a space larger than the limb bud itself (using the immersed-boundary method to represent the limb within the larger space [22]) or representing the 2D shape of the limb bud itself with an irregular triangular mesh, which can be used as the framework for a finite element method [32] or a spring-lattice method [23]. For our 3D model we used a tetrahedralised mesh to approximate the geometric domain. We used NetGen [28] to transform the closed 3D iso-surface *S_t0_* into a fully tetrahedralised mesh suitable for the FEM simulation (Figure 2G). A variety of meshes were generated with different spatial resolution (defining how fine or coarse the mesh is), and from these we chose a 3D mesh with approximately 6,000 vertices and 27,000 tetrahedrons, as a balance between computational expense and accuracy.

Philips et al. reported that vertebrate mesenchymal tissue behaves like an elastic solid over very short time scales but displays a liquid-like characteristic in response to stress in long-term culture [33]. The first FEM of limb development (2D) therefore employed the Navier-Stokes equations to represent the mesenchyme as a viscous incompressible fluid whose volume increases corresponding to a distributed source term, ***s***, which represents the patterns of cell division [22]. Due to the small size of the limb bud, and the extremely low velocities involved (∼75 µm/h, described above) we chose a modified version of the Navier-Stokes equation to describe the movement of mesenchyme in our 3D model, in agreement with other cases where convection is negligible [32],[34],[35]. This also agrees with the recent lattice model proposed by Morishita [23]. We have also performed comparative simulations with and without convection to demonstrate that it has no significant effect on the results (unpublished data). We used the following equations which describe the balance of forces acting at any given region of the fluid as follows:
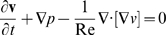
(3)

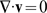
(4)where, *v* is velocity, *p* is pressure, and Re is the Reynolds number. Equation (4) describes fluid continuity, which usually represents the conservation of mass. In our case (following Dillon and Othmer [22] and Murea and Hentschel [32]) we alter this equation to allow a distributed material source term ***s*** which can vary arbitrarily across space and time:
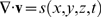
(5)In this way, the ***s*** field (tissue growth) can drive the velocity (tissue movements) of the system. Positive ***s*** values result in tissue growth at the position *x*,*y*,*z* at time *t*. In fact, ***s*** represents the proportional volumetric growth per unit time (otherwise known as the growth constant *k*, or the growth-frequency), so a value of 0.1 h^−1^ means that the volume expands by 10% in 1 h.

An important question is how to relate ***s*** to real cellular activities. We consider cell density (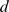) to be the number of cells (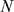) per unit volume (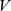):
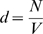
(6)The volumetric growth rate (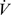) for a given region of tissue can therefore be completely defined as a function of two variables: the rate of cell number change (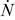) and the rate of cell density change (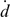). At some stages of development these two factors effectively cancel out; for example the first few rounds of zygotic cell division simply divide the existing cellular material into a larger number of smaller regions (cells). In other words, as the cell number increases, so does the cell density such that overall volume remains unchanged. By contrast, in limb development cell density does not change much during our 6 h time interval, such that growth is mostly driven by cell proliferation. It is also known that programmed cell death only occurs in three small well-defined regions in the limb buds of both mouse and chick [13].

For simplicity, we will start by assuming that *d* is constant. In this case 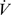 is proportional to 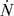, and ***s*** is equal to ***s_p_***, which we define as the proportional growth rate due to proliferation alone. Constant ***s_p_*** describes exponential growth, and in general then, the number of cells *N_t_* at a time point *t* can therefore be calculated from the number of cells at an earlier timepoint *N_0_* by the following equation:
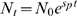
(7)When *t* equals the cell doubling time (*Tc*), then *N_t1_* must be double the value of *N_to_*; therefore ***s_p_*** can be calculated from *Tc* for each region of tissue as follows:
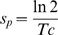
(8)It can be seen from this equation the intuitive fact that ***s_p_*** is inversely proportional to *Tc*—as the cell cycle becomes longer (slower), then growth rate decreases. Using equation (8) we can therefore approximate a 3D field of ***s_p_*** values that represents tissue growth. If we use hours as the unit of time, a cell cycle time of 10 h translates into an *s_p_* value of 0.07 h^−1^.

We next wished to check whether cell density changes significantly during the 6 h period between *t_0_* and *t_1_*. Analysis of nuclear-stained sections from four stages of limb bud development showed that although there are no spatial variations in cell density (mesenchymal condensations have not yet formed) there is a small but clear increase in the uniform cell density over time (Figure S2). From E11.0 to E11.25 the cell density increases at ∼1.7% per hour, which can therefore be represented by ***s_d_*** = 0.017 (the proportional rate of volumetric change due to cell density changes). An increase in cell density means that the cells are packed closer together (either due to a reduction in secretion of extra-cellular matrix (ECM), or a reduction in average cell size), and this therefore leads to a decrease in volume. The overall proportional volumetric growth rate is therefore defined as follows:
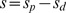
(9)In other words, if the proliferation causes volumetric expansion of 10% per hour, and density increase causes volumetric shrinkage of 1% per hour, the net growth will be 9% per hour.

We used the commercial software package FastFlo to solve these equations [36] and employed the artificial compressibility method to derive a solvable equation for pressure [37]. The suitability of this approach was tested using simple geometric figures (spheres) with a variety of source terms to confirm that the correct result was computed. The domain was implemented as a Lagrangian mesh—in other words the 3D mesh grows in unison with the growth of the limb bud. Each tetrahedron therefore represents a given piece of growing tissue within the bud. Although each of these tissue regions increases in size over the 6 h, we assume that the ***s*** value for each region is constant during the 6 h period (as justified above) and is thus carried along with the vertices of the mesh.

### Cell Proliferation Rates Cannot Explain Limb Bud Morphogenesis

After generating the two sets of quantitative 3D data on shape *S_t0_* and *S_t1_* and constructing the FEM of tissue growth we could start exploring the “*growth-based morphogenesis*” hypothesis. The 3D distribution of the source term ***s*** was directly calculated from our atlas of cell cycle times (using equations 8 and 9), and from *S_t0_* the simulation was run forwards over a period of 6 h to determine the new predicted limb bud shape *pS_t1_*.

Solving the equations requires a value for the Reynold's number, and previous studies have chosen a single value (equivalent to the viscosity of water) to explore limb growth dynamics [22]. However, the real effective viscosity of mesenchymal tissue is not known, and we therefore chose to explore a wide range of values, to ensure that our conclusions would not be dependent on this unknown parameter. We performed 6 simulations, covering 5 orders of magnitude from 10^−1^down to 10^−6^ (a viscosity similar to honey). Results of these simulations revealed that viscosity had only a minor impact on the final shape (Figure S3). Over the 5 orders of magnitude in range of Re explored, limb bud elongation varied by just 9% points. The real limb shows an outgrowth of 43% whereas the simulations showed an increase in PD length of between 3% and 12%. Thus, we conclude that the exact value of the viscosity used in these simulations is not a critical parameter.

**Figure 4:**
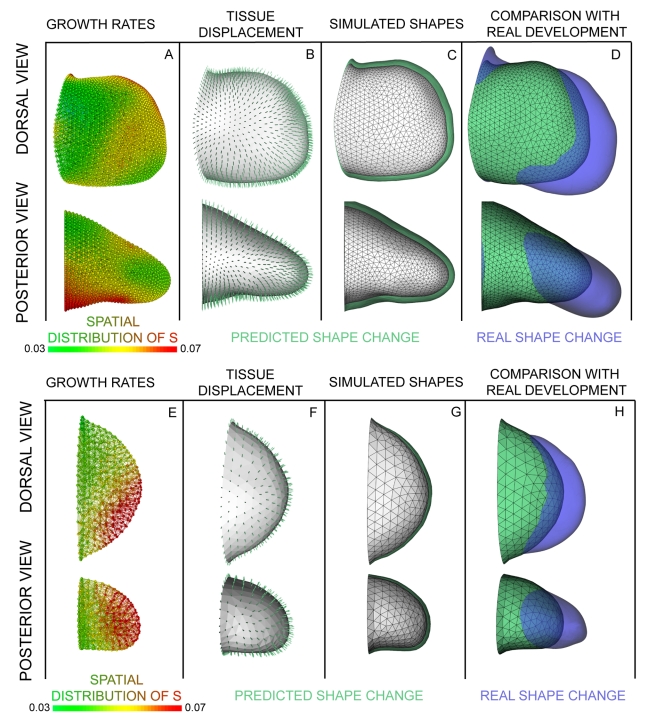
Numerical simulations based on empirical proliferation rates. Detailed analysis of the simulation with Re = 10^−2^. (A) The initial limb shape *S_t0_* with the measured proliferation pattern converted into source term. High proliferating zones can be found ventrally and distally, as well as a small area dorsally. Medium levels of proliferation can be found distally and ventrally, and a core of low proliferation centrally at the proximal end. (B) The tissue displacement calculated from (A) (green arrows) is fairly uniform surface expansion, resulting in an increased size rather than a shape change. (C) The initial shape *S_t0_* (white surface) was grown in silico into the predicted shape *pS_t1_* (green surface), which is larger but shows the same shape. (D) The predicted result *pS_t1_* (green surface) shows significant differences with the empirical measured limb *S_t1_* (blue). (E–H) show the same as above for a simulation of a younger E10.5 limb. Despite the simpler limb bud shape (compared to E11.0) this simulation also fails to generate the correct shape change.

The predicted shapes are all similar to each other (Figure S3), and none of them match the empirically measured *S_t1_*. A detailed analysis of one of these simulations (Re = 10^−2^) is highlighted in Figure 4A–D. Rather than a distally oriented outgrowth, the virtual limb bud shows fairly uniform growth in all directions (green arrows in Figure 4B) resulting in a predicted shape (green surface in Figure 4C,D) which is unlike the real measured shape (blue surface in Figure 4D). In order to confirm the general importance of this conclusion for limb bud development, we also repeated the entire analysis for a younger stage of hindlimb: 3D imaging by OPT, BrdU/IddU analysis, cell density counting, and finite element modelling was performed for an earlier limb bud shape (E10.5 growing to E10.75). As before, the proliferation pattern shows a slight gradient of values along the PD axis (Figure 4E) which in principle could agree with the “*growth-based morphogenesis*” hypothesis. However, the simulations using these empirical values confirmed again that the correct shape cannot be achieved using this model (Figure 4F–H).

In both simulations the final predicted volume of the limb bud is smaller than the real volume (a 15% and 21% deficit for the younger and older simulations, respectively). This is most likely explained by the fact that some cells are still entering the limb bud from the main body flank during early developmental stages.

Although we are confident of the estimate of *Tc* values, we also wished to double-check that a hypothetical error in our *Tc* calculation method could not account for the failure of elongation. We therefore performed an extra pair of simulations in which the estimated ***s*** values were uniformly scaled up such that the final volume equalled the real volume. This required average *Tc* values of 8.1 h and 9.5 h, respectively, for the younger and older simulations. Both of these “volume-corrected” simulations show the same result as before—a general increase in limb bud size in all directions, rather than distal-specific elongation (Figure S4). Additionally, we performed one final test, in which the average measured *Tc* value was distributed uniformly across the limb bud. The resulting shape was visually indistinguishable from the simulation using the measured gradient of *Tc* values, highlighting that this observed proliferation gradient has no significant effect compared to a flat distribution.

One way that a general isotropic increase in volume could be converted into a distal elongation is if the mesenchyme was “squeezed” by the ectoderm, and this idea has been proposed a few times [11],[22],[38]. This concept predicts that the mesenchyme exerts an outward force, which is mechanically resisted by the ectoderm. The strongest evidence against this idea was first described in the classical work of Saunders [20], in which he showed that removal of large segments of the dorsal ectoderm (up to three-fourths of the dorsal surface) did not interfere with relatively normal limb development—in particular that the mesenchymal tissue did not spill out through the ectodermal hole. This was later re-confirmed by Martin and Lewis [21] in experiments which specifically destroyed the dorsal ectoderm of the chick limb bud with UV radiation, while leaving the AER intact. We have also made similar observations in mouse limb buds grown in in vitro culture (Figure S5), and we therefore rule out the possibility that distal elongation is the product of an isotropic mesenchymal expansion being squeezed and restricted by an external ectodermal force.

Taken together our simulation results strongly suggest that in the absence of any directional cell behaviours the empirically measured proliferation pattern cannot produce the correct limb bud shape. Although this spatial pattern is apparently controlled quite precisely [13], with lower rates proximally and higher rates distally and near the ectoderm (Figure 3F), nevertheless this spatial gradient does not appear to be important for limb elongation.

### Exploring the Parameter Space of the Model

Our data-driven simulations strongly suggest that isotropic growth can be ruled out as the main force for distally directed limb bud elongation. However, despite our confidence that these are the most accurate empirical data sets generated for the limb bud so far (on 3D shape change and cell cycle times), nevertheless the possibility for some numerical errors remains. As our simulations depend heavily on the numerical details of these data sets, it is therefore essential to perform a systematic exploration of the parameters of the model: Within which bounds will our conclusions hold true? In other words, could a different set of isotropic growth rates be consistent with *growth-based morphogenesis*? Are there in fact multiple growth patterns that could be compatible? If so, how different would these proliferation rates be to our measured data?

To answer these questions we formulated our model as an inverse question. Rather than starting with the initial shape *S_t0_* and the growth data *T_C_* and asking what shape *pS_t1_* they predict (as done in the previous section), we start with the initial and final shapes (*S_t0_* and *S_t1_*) and ask the computer to search for a theoretical growth pattern which could produce this result. The inverse approach has previously been used for other complex developmental questions, such as deducing gene network design from the resulting expression patterns in *Drosophila* [39],[40]. The approach requires three components: (i) a parameterisation of the problem, (ii) a fitness function, and (iii) a method to find optimal solutions to the problem.

**Figure 5:**
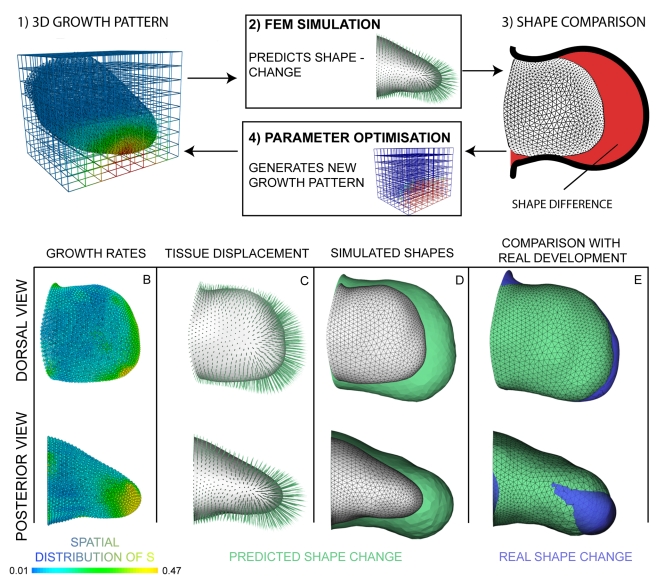
Exploring parameter space. (A) The optimization process is a loop where (1) the proliferation values are interpolated onto the limb mesh, (2) the simulation predicts a new shape *pS_t1_* according to the proliferation values, (3) the fitness function compares the predicted shape *pS_t1_* with the real shape *S_t1_*, and (4) the parameter optimization adapts the parameters according to the results of the shape comparison. This process is repeated (based on a Hooke and Jeeves direct search method [43]) until a stable proliferation pattern is obtained. (B) The proliferation pattern for positive isotropic growth shows low proliferation dorsally and ventrally, while high proliferation can be seen along the distal ridge with a trend towards the posterior side. (C) Directed tissue displacement in the PD direction is the result of the high proliferating in the distal zone. (D) Compared to the *S_t0_* limb shape (white), a clear shape change is evident for *pS_t1_* (green). (E) Comparing the predicted limb shape *pS_t1_* to the empirical measured *S_t1_* shows a high degree of similarity, but still overextended outgrowth, especially along the DV axis. Limb orientations are as shown in Figure 2F.

- The parameters we explore are the growth values in different regions of the limb bud. For an optimisation project, this is a novel type of parameter compared to previous studies—rather than global constants of the system (for example the Reynolds number of the tissue or a gene interaction strength [39]) here we wish to optimise a spatially distributed set of local values. We therefore had to choose a parameter set which could define any arbitrary 3D distribution of scalar values—a new challenge in the field of biological model optimisation. To minimise the constraints on these spatial patterns, we first explored the simple idea of assigning an independent growth parameter to every vertex of the tetrahedral mesh. Although successful, this method was inefficient—T_c_ values always vary smoothly over space (Figure 3B,F), and therefore significant redundancy is introduced by employing a parameter for each of the 5,959 vertices. We therefore sought a spatially coarser parameterisation method and implemented a regular, orthogonal grid which is superimposed into the same 3D space as the limb mesh (Figure 5A and Video S1). The spatial resolution of the grid can be adjusted independently of the tetrahedral limb mesh, and the full smoothly varying growth pattern can therefore be defined by a lower number of parameters (assigned to the vertices of the cube-mesh and then interpolated onto the limb mesh). For this study we chose a mesh in which 525 vertices define the full 3D pattern for the limb bud (see Methods for more details).
- The fitness function (or objective function) should be a single value which indicates how good a simulation is, thereby guiding the optimization process to find the best result. In our case, the fitness function is a measure of *shape difference* between the computer prediction *pS_t1_* and the real limb shape *S_t1_* (Figure 5A). As this value tends to zero the two shapes converge, implying a perfect solution. We implemented a fast shape-difference estimator by summing the absolute distances of every surface-vertex in the predicted shape to the closest surface-triangle in the real shape.
- Parameter optimization can be described through the concept of a fitness landscape (by analogy with a real mountain landscape). The full space of the landscape represents the full range of parameter combinations (i.e. the range of possible 3D growth patterns), and the height of the landscape at any given position represents the fitness of those particular parameters. The algorithm can only determine the fitness of a given point by performing a complete simulation with those parameter values, but a comprehensive survey of the whole landscape is far too computationally expensive (many millions of simulations would have to be performed). The goal of an optimization method is therefore to find the best solution by a series of carefully selected simulations, thereby exploring its way through the landscape towards the higher mountains. (In practice our fitness function is the shape difference, and it therefore *decreases* in value as the fitness improves. We describe the optimization analogy here as an upward hill-climbing process merely as the usual convention to illustrate the concepts.)

In general, two classes of optimization methods are distinguished: local and global (recently reviewed in Ashyraliyev et al. [41]). Global search methods are necessary when the search space is likely to have many fitness optima (known as a rugged landscape), making it hard to locate the true global best result. They mostly employ stochastic functions to avoid getting trapped in local optima (for example Simulated Annealing [42]). Local optimization methods by contrast can be used for either low dimensional or constrained problems (known as a correlated fitness landscape). Local methods start from a specific initial set of parameter values (i.e. an initial position in the landscape) and assume that a continuous “uphill” path leads to the global optimum.

Theoretical considerations suggested that our choice of parameterization (defining an independent growth value for each region of tissue) would create a smooth, correlated landscape. We therefore explored the use of a local search method. (This choice is verified in the next section.) Due to the rather slow running time of a single simulation (5–8 min) we chose the Hooke and Jeeves direct search method (rather than a gradient-based method) [43]. At each iteration of the optimization, all 525 parameters are tested individually to assess whether a small local increase or decrease in the growth rate improves the solution. At the end of each iteration a new candidate solution is constructed by combining all the individual improvements (thereby implementing a diagonal move in parameter space). The magnitude of the increments/decrements is reduced during the course of the optimization process to allow finer adjustments as the solution improves (similar to the cooling schedule employed in Simulated Annealing [42]).

### *Growth-Based Morphogenesis* Is Theoretically Possible But Not Realistic

Once developed, we used this optimization strategy to ask if a 3D proliferation pattern could be found that would give us a shape similar to the empirical measured *S_t1_*—in other words, is it at all possible that a 3D distribution of purely isotropic behaviours can explain normal limb bud development? We restricted the optimized ***s*** term to be positive, so that despite the belief that some cells enter into the limb bud from the flank, it is nevertheless reasonable to compare the optimized ***s*** values to the results from our BrdU/IddU double-labelling experiments. Other possible influences like cell death were thus intentionally neglected. After 75 iterations of optimization a shape was produced which showed a significantly higher similarity to the *S_t1_* shape than our previous result (using the empirical BrdU/IddU data Figure 4), although the DV and AP growth was still greater than the real *S_t1_* (Figure 5B–E). This improved shape change was explained by a dramatic difference in proliferation pattern. This optimized pattern shows a much stronger spatial gradient along the PD axis—with ***s*** ranging from just above zero in much of the bud to 0.47 h^−1^ in the distal-most regions (Figure 5B). This contrasts with the empirical cell cycle data which range from s = 0.03 h^−1^ to just 0.07 h^−1^ (Figure 4B).

Since the resulting simulated shape still did not match the empirical *S_t1_* we next asked a more general question. Rather than restricting the exploration of ***s*** to positive values—which could correspond specifically to proliferation (equation 7)—we extended the possible range of ***s*** into negative values, thereby allowing active tissue shrinkage to also play a role, which in principle could occur by programmed cell death or local increases in cell density. In practice this allows ***s*** to represent the combined effect of any isotropic cell behaviours. Adding this change and rerunning the optimization achieved a large improvement in the match between the shapes of *pS_t1_* and *S_t1_* (Video S2). However, before analyzing this result in detail we wished to confirm that our choice of a local search method was suitable for this problem—in other words to determine whether this optimization process would become trapped on local optima rather than finding a genuine global result.

**Figure 6:**
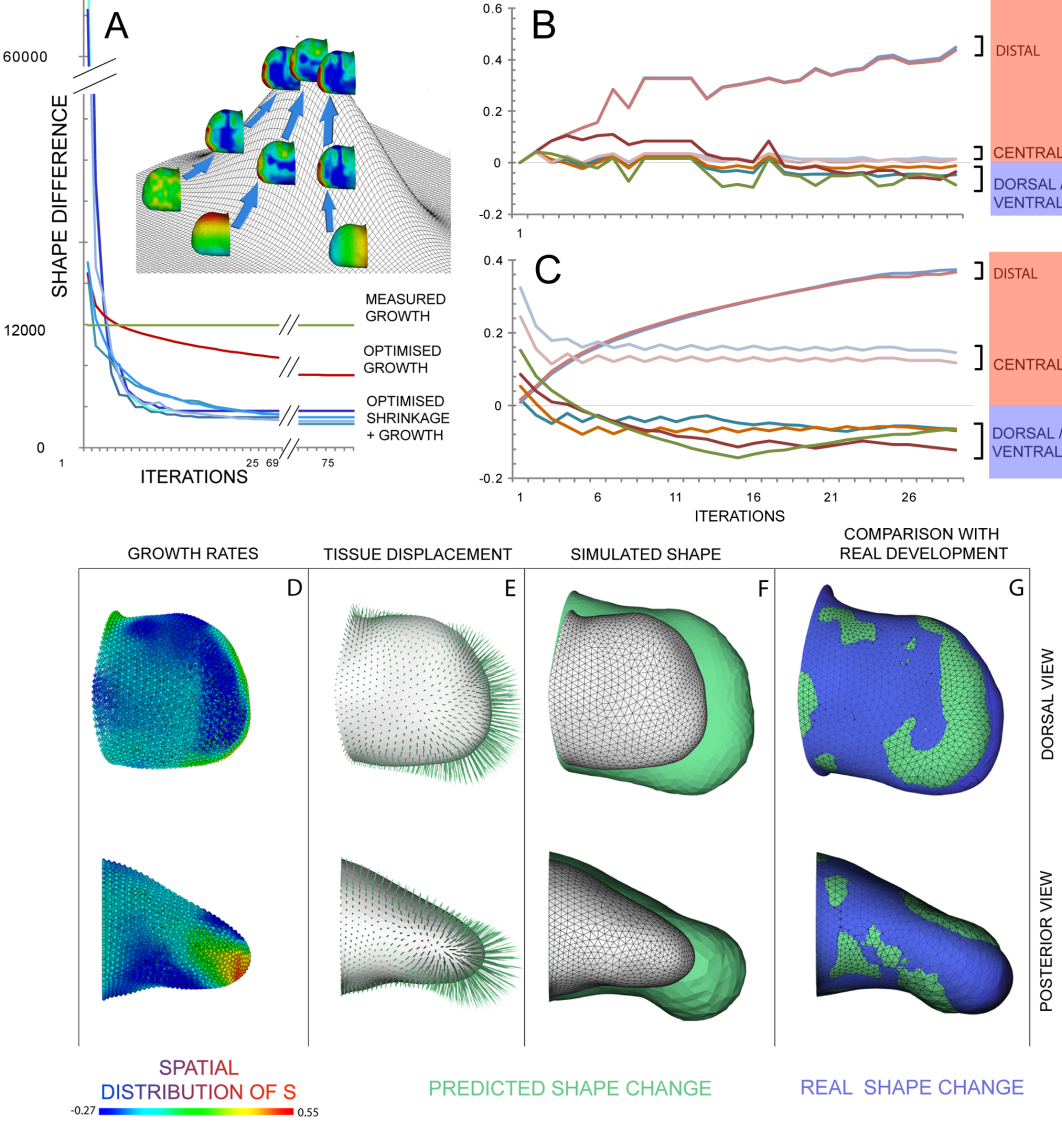
Assessing the parameter optimisations. (A) A plot of the fitness improvements (decrease in shape difference on the *y*-axis) against the successive iterations of the optimisation process (*x*-axis). At each iteration the shape difference decreases until it converges to a stable minimum. For comparison, the green line shows the difference between *S_t1_* and *pS_t1_* when using the BrdU/IddU data (i.e. not optimised). The red line shows the result of the optimisation with only positive proliferation (Figure 5B–E), which achieves a lower shape difference. The blue curves are the results of optimisations which allowed negative *s* values (tissue shrinkage). The multiple blue lines represent optimisations starting with different initial proliferation patterns, and all converge to similar results which are ∼70% lower (better) than the BrdU/IddU data (green line). Although the optimal shape difference was the minimal difference, we have illustrated the global optimum of the landscape as a high peak, following the convention of the hill-climbing analogy. Starting from different points of the fitness landscape, the parameter optimisation converges to the same “mountain peak” with the same basic pattern of growth values. In (B) and (C) the *x*-axis is the same as (A), but the *y*-axis plots the changing *s* values for different positions within the limb bud. Neighbouring pairs of vertices are tracked for each of four regions (distal, central, dorsal, and ventral) and illustrate that although the two cases start with different distributions of initial *s* values, these parameters converge to the same general layout, with high growth in distal regions, low in central regions, and tissue shrinkage in dorsal and ventral regions. (D–G) shows the results in more detail for one of the optimisation runs. The final growth pattern (D) clearly shows a discrete region of very high proliferation at the distal tip (red/yellow) and shrinking areas dorsal and ventrally (blue). The resulting tissue displacements (E) generate a new shape *pS_t1_* (green surface in F,G) which is much flatter than before and shows a close correspondence to the real shape *S_t1_* (blue in G). Limb orientations are as shown in Figure 2F.

A common strategy to address this question is to start the simulation at multiple different initial conditions (i.e. different positions in the landscape) and explore whether it always converges to similar solutions. In addition to the two previous optimisations (which were started with all ***s*** values set to zero) we chose five additional initial conditions—two different linear gradients of proliferation in different directions, two radial gradients (either increasing or decreasing from the centre) and one pattern with a random distribution of values. These initial patterns were chosen to represent a collection of extreme alternative spatial distributions (Figure S6), thereby covering a wide region of parameter space. These tests all resulted in a very similar final optimized distribution within about 25 iterations. Plotting the shape difference over iterations of the optimisation process shows all five cases converging rapidly to a good fit (blue lines in Figure 6A). Interestingly, they converged faster than the previous case (when ***s*** values could not go below zero), which required 75 iterations before stabilizing (red line in Figure 6A). Examination of the final patterns showed that despite some small variations, they had all converged on the same solution (see also Figure S6), strongly suggesting that the fitness landscape for this problem contains one global optimum, which is reachable along a continuous path of incremental fitness improvements from many different starting positions.

To further verify this conclusion we monitored the change in ***s*** values during the optimization process at four specific positions within the limb bud: distal, central, dorsal, and ventral. In the first example all ***s*** values started at zero (Figure 6B). During optimization the ***s*** values for distal tissue increased up to around 0.4 h^−1^, central values increased a small amount, and dorsal/ventral values decreased to negative values. In the second example, the initial pattern of ***s*** values displayed a variety of different values for different regions—distal tissue started with low values, and central tissue with high values. During optimization, these values “crossed over” to converge on a final pattern similar to the previous case (with high distal values around 0.4 h^−1^, medium central values of around 0.15 h^−1^, and again negative values for dorsal/ventral tissue). These results strengthen the conclusion that one global optimal solution exists which is repeatedly found, irrespective of the starting values.

One of these optimization results is shown in more detail in Figure 6D–G. The much closer correspondence of the limb bud shapes can be seen by the more complex intersection between the green and blue surfaces in Figure 6G (representing the predicted and real shapes, respectively), as compared to Figures 4D and 5E. More specifically, the shape comparison is ∼70% better than the shape produced by the real BrdU/IddU data (blue graphs compared to green line in Figure 6A). This is only achieved by having very high proliferation in a very narrow distal region and negative values in much of the mesenchyme. In fact 22.5% of the volume of the mesenchymal tissue adopts a negative ***s*** value (all the blue regions in Figure 6D), suggesting that if cells only perform isotropic activities, significant tissue shrinkage would be necessary to achieve the correct shape (more than 10% shrinkage per hour in large regions of the bud).

The primary motivation for this parameter exploration was to determine whether numerical errors in our cell cycle data could account for the model's inability to generate the correct limb bud shape. The optimization experiments produced two main results. (1) The concept of *growth-based morphogenesis* is theoretically possible—a pattern exists (Figure 6D) which can indeed produce the observed shape changes. (2) However, this pattern is dramatically different from our measured BrdU/IddU data (Figure 4B), both quantitatively and qualitatively. For example, in the optimised result, the volumetric growth in the distal region must be as high as >0.5 h^−1^. Since cell density does not significantly change over these developmental stages (described above), the major source of volumetric growth is indeed cell division, and the model would require a cell cycle time of less than 1.5 h, which has never been observed in the growing mouse limb bud. Additionally, the predicted regions of strong tissue shrinkage cannot be explained by programmed cell death, as this is well known to occur in just three small regions [13], rather than the large predicted 22% volume of the limb bud. At a more general level we have shown that purely isotropic cell behaviours (such as cell proliferation, cell death, and change in cell density) can be ruled out as the driving force for limb bud outgrowth.

### Mesenchymal Cells Display Complex, Highly Dynamic 3D Shapes and a Strong Orientation Bias

Our computer modelling makes the prediction that correct limb bud morphogenesis requires some kind of directional cellular activities. To verify this prediction we went back to the limb bud, to search for evidence of oriented cellular structure within the mesenchyme. Determining the shape of individual cells can be facilitated in two ways: (a) Membrane-specific labels dramatically increase the chance of visualizing the fine-structure of the cell outline. Cytosolic labels by comparison tend to produce an intense signal from the main cell body that outshines fine details of the membrane. (b) However, labelling too many adjacent cells can make it impossible to delineate each one clearly, and so membrane dyes such as bodipyceramide are unhelpful. The ideal approach labels the membranes of just a small subset of cells within an unlabelled tissue, thereby allowing each cell shape to be highlighted precisely. We therefore chose to electroporate a membrane-targeted GFP construct into the lateral plate mesoderm of HH15 chick embryos in ovo, to achieve stochastic cell labelling in the limb bud mesenchyme 24 h later (HH21).

**Figure 7:**
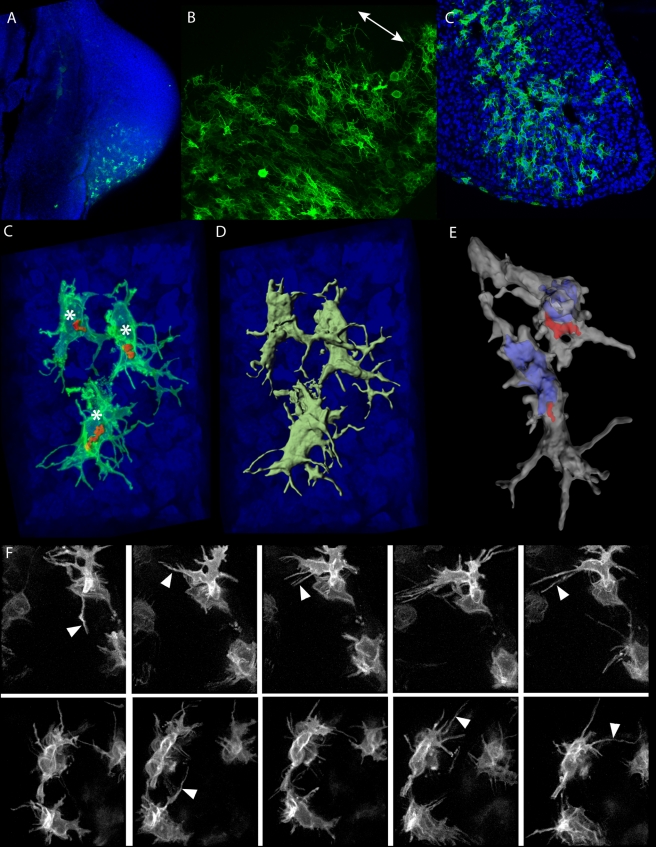
Limb bud mesenchymal cells display complex 3D shapes with highly dynamic filopodia. (A) Overview of a typical in ovo electroporation result. All cell nuclei are labelled with DAPI (blue) and the GFP expression can be seen in green. (B) When many cells are labelled a general alignment of cellular processes is evident, oriented perpendicular (white double arrow) to the nearest ectoderm (bottom-right). (C) A section through the middle of a chick limb bud, showing the complex, branched and extended morphology of almost all the randomly labelling cells. (D–E) High-magnification 3D images of a few labelled cells, showing how the Golgi (red) is consistently on the same side of the nucleus (asterisks in C, blue shape in E) and revealing the extended cellular processes of the complex 3D geometry of each cell. (F) Frames from two time-lapse movies showing the dynamic extension and retraction of the filopodia (white arrowheads) over a 2 h period.

Confocal microscopy of labelled cells revealed a striking morphology. The vast majority of mesenchymal cells have very complex 3D shapes, exhibiting long filopodial processes which branch extensively and extend up to 3 cell diameters away (Figure 7A–C). These shapes do not strongly resemble the “classical” 2D migrating cell morphology with a broad leading edge and narrow trailing edge, although this could be due to the genuinely 3D nature of the mesenchymal environment—indeed for some cells the Golgi-side appears to have more filopodia than the opposite side (Figure 7C–E). Although an unambiguous orientation is not clear for individual cells, when many adjacent cells are labelled a gross orientation of the cellular processes can be discerned (double-headed arrow in Figure 7B). This rough orientation is towards the nearby ectoderm and therefore perpendicular to the main PD axis, rather than towards the AER, possibly suggesting a cell intercalatory mechanism rather than a simple distal-wards migration. Apart from the extensive filopodia, we failed to detect another type of cellular process that has previously been described for limb bud mesenchymal cells: cytonemes, which are long processes (up to 700 µm) which are much thinner than typical filopodia and with a constant cross-sectional profile approximately 0.2 µm across.

Since the complex 3D distribution of filopodia could be the driving force behind cell migration or intercalation, we performed time-lapse imaging of the electroporated limb buds in ovo, to determine how active they are. This imaging revealed a highly dynamic nature of the filopodial protrusions—contracting and re-extending in a manner reminiscent to migrating fibroblasts (Figure 7F). Considering that almost all labelled mesenchymal cells display this complex array of dynamic filopodia, these observations considerably alter the classical view of the limb bud mesenchyme which has been repeatedly modelled as if cell divisions and cell growth represented the main source of force-generation [22],[23],[25],[32]. As before, a single preferential direction for filopodial activity was not evident, arguing against a simple model of chemotactic migration towards the AER.

**Figure 8:**
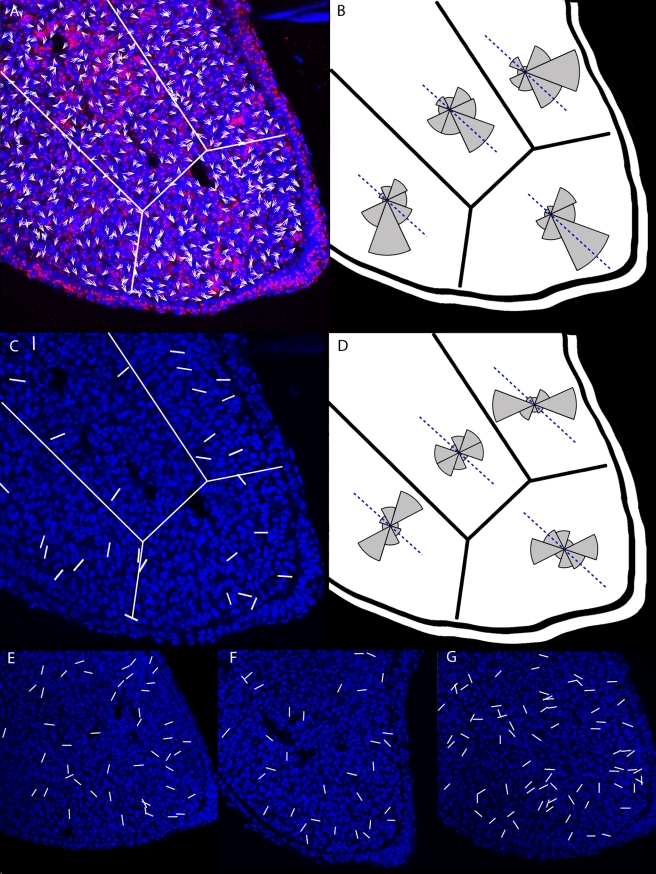
The Golgi and cell division orientations of limb bud mesenchymal cells. (A) A map of Golgi body orientation (relative to the nucleus) on cells across the same transverse section as Figure 7C. (B) A summary of the orientation bias seen within four different regions of the mesenchymal tissue (*n* = 565). Dashed blue line indicates the main PD axis of elongation. (C) A map of cell division orientation; the ends of the white lines indicate the positions of daughter chromatids during telophase. (D) A summary of the bias in cell division orientation. Since each section contains a limited number of cells in telophase, these results (*n* = 187) were collated from all four sections seen in (C) and (E–G).

To further explore the possible orientation of these cells we labelled the Golgi apparatus, because in many actively migrating cells it displays a biased position between the nucleus and the leading edge [44]. Golgi orientation in the developing limb bud mesenchyme has previously been explored with respect to the ectoderm [45] and the developing mesenchymal condensations [46],[47]. Interestingly, whereas a slight bias towards the ectoderm was previously reported at later stages of limb development (>E12.5 in the mouse or HH24 in chick), we already see a clear bias at HH21 (Figure 8A,B). In more than 70% of cells the Golgi is positioned on the distal side of the cell (*n* = 565). Interestingly, although Golgi orientation shows more of a distal bias than the general cell shape, it is also not aligned strictly towards the AER—it appears to be influenced both by the AER and by the nearby ectoderm. In other words despite a general distal-wards bias, we do not find a precise alignment between Golgi position and the direction of limb elongation. If cell orientation is an important aspect of normal morphogenesis, then it is possible that the presence of cells pointing in the opposite direction is the consequence of cell divisions, during which the daughter cells must at least temporarily have their Golgi on opposite sides of the cell (due to the movements of spindle formation and chromosome segregation). This would effectively reduce the strength of the measurable orientation bias.

Although a previous study had reported a lack of bias in the orientation of cell division [11], our interest in possible directional cell behaviours prompted us to re-evaluate this question. By measuring the angle of the relative positions of daughter chromatids at telophase (*n* = 187) we revealed that there is indeed a clear bias in the cell division orientation in different regions of the limb bud (Figure 8C–G). In regions close to the ectoderm this bias is similar to the bias for Golgi positions (partly distal and partly ectodermal). Indeed as mentioned above, these two observations could be linked by the fact that during cell division Golgi positions will be determined by the positions of the daughter cells. However, in the central region of mesenchyme furthest from the ectoderm, this correlation is not seen—Golgi bias is clearly distally oriented, while the cell division shows a slight bias perpendicular to this (i.e. along the DV axis). We also found a bias in cell division orientation of mouse limb bud mesenchymal cells, by tracking the angles of cytokenesis in time-lapse confocal movies of mouse limb buds cultured in vitro (Figure S7, and methods in Text S1).

## Discussion

### The Predictions of *Growth-Based Morphogenesis* Are Inconsistent with Empirical Data

The cells in a developing organ can perform a wide range of active behaviours and movements—for example cell division, cell death, secretion of ECM, changes in cell size, active migration, intercalation, and convergent extension. A central difficulty for biologists is to pinpoint which behaviours are responsible for tissue-level movements. With state-of-the-art time-lapse microscopy it is increasingly possible to watch these behaviours directly, but observing a behaviour does not prove that it has a role in generating the tissue-level forces. For example, cell intercalation can either be the driving force behind convergent extension [48] or can alternatively be the by-product of tissue movements driven by other external forces. While a few previous studies on the limb have sought to uncover which cell behaviours might contribute to limb bud outgrowth [11],[19], here we have chosen a very different approach—to focus on the most popular concept, *growth-based morphogenesis*, and to rigorously test its sufficiency as a mechanistic explanation.

The concept of *growth-based morphogenesis* is both an intuitive and popular explanation for limb bud outgrowth [9],[10],[23]. Even studies which revealed a possible alternative force for outgrowth still refer to the need for high proliferation localized specifically in the progress zone, stimulated by the mitogenic effects of FGFs secreted from the AER [19]. Similarly, studies which revealed the proliferative influence of ectodermal WNTs across the whole limb bud still also claimed a need for higher proliferation in the progress zone due to the combined effects of general ectodermal signals (WNTs) and specific AER signals (FGFs) [24]. The idea has twice been translated into a formal computer model [22],[23], however in both cases the idea was only explored at a conceptual level. Both models operated in 2D rather than 3D and did not attempt to integrate empirical growth data. By contrast, through novel image processing techniques we have measured the variables most relevant for the concept (3D shape changes and proliferation rates) and directly tested whether the theory is compatible with reality. In particular, this was achieved by introducing a novel approach for parameter optimization—rather than optimizing a low-dimensional space of global parameters, we efficiently explored a 525-dimensional parameter space of local growth values, thereby creating quantitative predictions about how the *growth-based morphogenesis* concept could be theoretically possible (Figure S9). Interestingly, our data-driven growth model is effectively an extension of a 1D model produced over 30 years ago by Lewis [49], in which he compiled the values of mitotic index along the PD axis and performed a similar growth calculation for the goal of creating a mathematical linear fate-map of the growing chick wing bud. It is the use of new data-capture techniques and finite-element modelling which has enabled us to overcome the challenge of extending his approach into 3D.

An intriguing finding is that there is indeed a reproducible way in which the theory can produce the correct 3D shape. The successful growth pattern displays a very specific spatial distribution, and furthermore, this optimized pattern directly reflects the general assumptions behind *growth-based morphogenesis*—that mesenchymal proliferation rates are highest close to the AER. The correspondence between the predicted stripe of high proliferation (red regions of limb buds in Figure 7A and Figure S6) and the location of the AER is striking, because no information about the ridge was included in the model. In principle, this result provides general confirmation that the underlying concept of *growth-based morphogenesis* is theoretically possible, as shown previously in 2D [22],[23].

**Figure 9:**
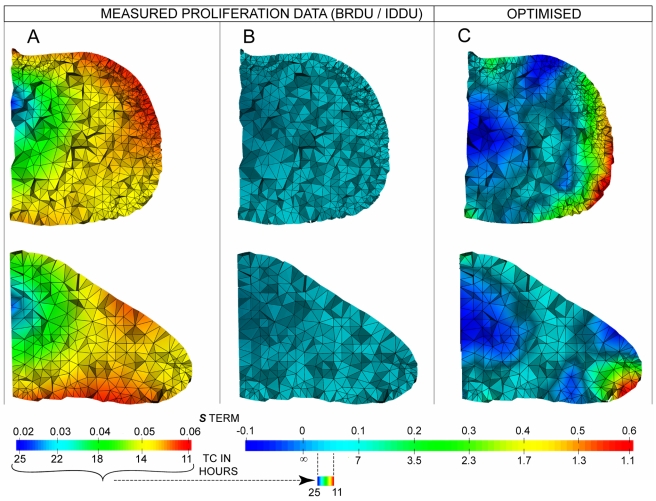
Comparing optimised versus real growth rates. Proliferation maps on two orthogonal virtual cross-sections through the limb, from measured data and optimized simulations. (A and B) both show exactly the same empirical data (the real cell cycle times), but with two different colour maps, whereas (C) shows the fully optimised result. In (A) the map has been normalized with respect to the fastest and slowest dividing cells (a cell cycle time of 11 h (red) and 25 h (blue), respectively) and reveals a core of low proliferation in the central proximal region and areas of high proliferation in ventral and anterior regions. In (B), which shows exactly the same empirical data as (A), the colour map has been normalized with respect to the fully optimsed result shown in (C) and in Figure 6D. (C) To achieve *growth-based morphogenesis* the proliferation rates must adopt extreme values—with *s* ranging from −0.1 h^−1^ to 0.6 h^−1^. When the real cell cycle times are displayed with the same colour map (B) they constitute a very narrow range between these extremes—hardly any variation is seen. This is also highlighted by mapping the colour map for (A) under the colour map for (B) and (C), where again it can be seen that the range of empirical values is a small fraction of the extreme values required for *growth-based morphogenesis*.

However, because our model is 3D and includes accurate information about the real shape of the limb, we can go a step further and compare the values of parameters in the model with real life measurements. It thus becomes clear that the growth pattern required to make the model work (extremely high proliferation just under the ridge, and negative values (tissue shrinkage) in a large proportion of the mesenchyme) is not reflected either in the quantification of real cell cycle times nor in the known small zones of programmed cell death [13]. Figure 9 highlights this comparison through the use of two different colour maps. Figure 8A shows the spatial pattern of real proliferation rates in two orthogonal cross-sections through the limb bud, with a colour map normalized with respect to the fastest and slowest dividing cells (a cell cycle time of 11 h (red) and 25 h (blue), respectively). Panel B shows the same information (the real cell cycle times), but with a colour map which has been normalized with respect to the fully optimised result from Figure 6D, which is shown in panel C. To achieve *growth-based morphogenesis* the proliferation rates must adopt extreme values—ranging from −0.1 h^−1^ to 0.6 h^−1^. If the real cell cycle times are displayed with the same colour map (panel B) they constitute a very narrow range between these extremes—hardly any variation is seen. This is reflected in the observation that this real but shallow gradient of cell cycle times (from 11 to 25 h) has no significant impact on limb bud shape but only provides an increase in overall size (Figure 4D). We therefore suggest that the major role of cell proliferation is simply to provide enough progenitor cells for the ongoing development of the limb and that the spatial pattern of proliferative rates is likely to reflect other constraints, such as the gradual condensation of skeletal elements in the core of the bud, which is known to correlate with reduced proliferation rates [50].

This study started with real laboratory measurements (on shape change and cell cycle times), combined them into a computational model, and then ruled out isotropic cell behaviours as a possible explanation for limb bud outgrowth. It thus makes a prediction which brings us full-circle, back into the lab in search for biological evidence of oriented cell activities. Our search has been fruitful in uncovering clear evidence for a variety of oriented cell behaviours (Figures 7 and 8). The complex, extensive, and dynamic cell protrusions have not been previously described in the limb and raise a number of fascinating questions. The cell shapes themselves, plus the dynamic extension and retraction of filopodia, are indicative of active cells which could perform migration or active intercalation. The biased orientation of filopodia, Golgi, and cell divisions is not strictly distal but does display some distal bias. As a proof-of-concept we created a final simulation in which a hypothetical distal “migratory” force was added to the outward force generated by the isotropic growth pattern, creating a hybrid force field which is partly distal and partly towards the ectoderm (reflecting our observations above). This resulting hybrid growth orientation was indeed able to generate a shape which resembles the real shape change (Figure S8).

Cell migration has typically been studied in 2D contexts, so one possibility is that migrating cells are present but hard to identify because the genuinely 3D environment obscures any obvious leading edge and trailing edge. However an alternative (which is not mutually exclusive) is that the ectodermal orientation of these processes reflects an intercalatory mechanism producing a convergent-extension mode of tissue movement. In this scenario cells would be selectively pulling against their neighbours in a plane perpendicular to the PD axis (convergence), and the resultant squeezing together would push cells out along the PD axis (extension). Unlike classical cases of convergent-extension [48], the convergence would be balanced by cell proliferation, such that the limb bud does not become narrower over time. The vertebrate limb may thus provide a new paradigm for understanding 3D collective cell movements which involves a complex balance of oriented cell activities. Another intriguing question is how cell mixing and/or migration can occur without the extended cellular processes becoming entangled. Interestingly, Figure 7B shows that a few cells in the field-of-view have rounded up into a spherical shape, in a manner suggestive of cell division. If cells retract their cellular processes every time they divide, as has been observed in many other systems, then this could resolve the problem of entanglement.

In conclusion, we provide both theoretical and empirical evidence suggesting that distal elongation is driven by directional cell behaviours, rather than the proliferation gradient hypothesis (or *growth-based morphogenesis*). Oriented cell activities are often controlled by the PCP system, and our prediction may therefore help explain the phenotype of *Wnt5a* mutants. WNT signalling is known to orient the PCP system in mesenchymal cells undergoing convergent-extension [51], and intriguingly the phenotype of Wnt5a knock-out mouse embryos includes reduced limb bud elongation [52].

## Material and Methods

### OPT Scanning

First the tissue is washed 3 times in PBS, for 10 min each time, to wash out excess fixative. It was then embedded in 1% LMP agarose made with sterile dH_2_O. When set, the agarose block was trimmed with a surgical blade, ensuring adequate agarose was left surrounding the tissue, and glued to a cylindrical metal mount in the desired orientation for scanning. The samples were then placed into a clean glass container (height 5.5 cm) and tissue dehydrated at room temperature overnight by the careful addition of 100% MeOH to cover the sample. The following day, three 10 min washes are performed using MeOH, which was then replaced with BABB (1 part Benzyl Alcohol (Sigma) to 2 parts Benzyl Benzoate (Sigma)). With the lid removed, the bijou bottle is allowed to stand overnight at room temperature in a fume hood, to allow any remaining MeOH to evaporate. Samples were usually kept in BABB overnight and scanned the following day.

### Proliferation Measurement

Pregnant mice were administered IddU followed by BrdU by sequential interperitoneal injections after a defined inter-injection interval (T_i_) of 150 min. Embryos were harvested and fixed 30 min later, as this is known to be long enough for BrdU to reach the mesenchymal cells from the maternal blood stream and incorporate into the replicating DNA [30],[53].

#### Histology

To facilitate close histological analysis of thin sections of limb tissue, the limbs were embedded in wax prior to microtome sectioning. Prior to wax embedding, tissue stored in 100% MeOH was given two 10 min washes in 80% EtOH/dH_2_O followed by two 10 min washes in 100% EtOH. This was followed by three 15 min incubations in Xylene, the first of which was at room temperature and the remaining two at 58°C. The embryo was then incubated in 50% Xylene: 50% wax for 15 min, after which it was put through three 20 min incubations in fresh molten wax at 58°C. The wax containing the specimen was then tipped out into a suitable mould. The embryo was orientated under a microscope using forceps and the wax was left to set. The wax block was then cut into a pentagonal shape conducive for sectioning.

Wax embedded samples were sectioned at 7 µm on a microtome and ribbons of sections were floated out in a 42°C water bath. The sections were transferred on to Superfrost Plus electro-statically charged slides (BDH) with the aid of a fine-tipped brush. The slides were air dried and then transferred to a 55°C oven to dry overnight which were then stored at 4°C.

#### Immuno-staining

For antigen retrieval, slides were left to sit at room temperature in 60 mM sodium citrate (pH6). The buffer was then boiled in the microwave (900W) for 30 min and then allowed to cool for an hour. Slides were removed from the buffer and placed in blocking solution (TBST, 0.05% Triton, 10% heat-inactivated sheep serum) for an hour at room temperature. Sections were then incubated in blocking solution containing rat anti-BrdU (1∶100; clone BUI/75 Abecam) and mouse anti-BrdU (1∶50; clone B44 from Becton Dickinson). Incubation was completed at room temperature for 30 min and then 4°C overnight, followed by a 1 h wash in TBST. Sections were incubated with secondary antibodies Cy3 (1∶200) and Alexa 488 (1∶200; highly cross absorbed goat anti-mouse IgG antibodies) at room temperature for 30 min and then 4°C overnight. Slides were rinsed for an hour using TBST and then mounted using vectashield mounting medium containing DAPI (1/600). Slides were stored in the dark at 4°C for a maximum of 4 wk, after which time the fluorescent signal is lost.

#### Microscopy

The wax sections were scanned using a Leica TCS-SPE confocal microscope with a 10×/0.3 ACS-APO AIR objective.

### Cell Shape Analysis

#### Electroporation

Fertilized eggs were purchased in a local farm and incubated on 38°C for 65 h. In ovo electroporation was carried out as previously described [54]. Briefly, HH15 eggs were windowed, the vitelline membrane was removed, and 1 ml of 0.01% Fast green (F2752, Sigma Aldrich) in PBS was added on top of the embryo for better visualization. 1 µl of 1% Fast green was added to 5 µl of pCAGGS-gpiGFP vector (kindly provided by Fernando Giraldez Orgaz) at a concentration of 4 µg/µl. The DNA solution was injected into the embryonic cleome with microinjector (PicoPump PV820, WPI) and a sharp pulled glass pipette. For electroporation, CUY-21EDIT electroporator (Nepa Gene, Ichikawa, Japan) was used. A platinum anode was inserted beneath the embryonic endoderm and a platinum cathode was placed above the ectoderm surface. Three pulses of 4V, 60 ms pulse-on, 50 ms pulse-off were applied immediately after DNA injection. A small amount of 1% penicillin-streptomycin-amphotericin B (A5955, Sigma Aldrich) in PBS was added before the eggs were resealed and re-incubated for 24 h.

#### Immuno-staining

Embryos were dissected and fixed overnight in 4% paraformaldehyde in PBS at 4°C, embedded in 5% low melting point agarose, and vibratome-sectioned at 200 µm. Sections were placed on Superfrost Plus glass slides and blocked overnight on room temperature (RT) in blocking solution: 10% heat-inactivated goat serum (G9023, Sigma Aldrich) in TBST (0.1% Tween 20 in 1×TBS) with 0.01% Natrium Azide. Sections were incubated for 24 h on RT in blocking solution containing mouse anti-GM130 (1∶250; 610822, BD Transduction Laboratories) and rabbit anti-GFP (1∶500; 632460, Clontech). They were then rinsed in TBST with 0.01% Potassium azide overnight on RT and incubated in blocking solution with anti-mouse Alexa 568 (1∶200; highly cross absorbed goat anti-mouse IgG antibodies, A11031, Invitrogen), anti-rabbit Alexa 488 (1∶200; highly cross absorbed goat anti-rabbit IgG antibodies, A11034, Invitrogen), and DAPI (1∶500; D8417, Sigma Aldrich) for 24 h on RT. Finally, slides were rinsed overnight on RT in TBST with 0.01% Potassium azide.

#### Imaging

Sections were scanned using Leica SP5 inverted confocal microscope with 40×/1.25 APO oil immersion and 63×/1.40–0.60 APO oil λ immersion objectives. For Golgi analysis and iso-surfaces, z stacks were obtained with 1 µm or 0.1 µm z steps, respectively. Golgi orientation was analyzed with ImageJ. Arrows were drawn from the center of the nucleus through the middle of the Golgi on the entire z stack. The stack was then maximum projected to visualize all the arrows. For cell division analysis angles were measured (using ImageJ) of lines joining the daughter chromatin centres at telophase. Imaris ×64 was used to create the iso-surface images.

Time-lapse in ovo imaging technique was adapted from Kulesa and Fraser [55]. Briefly, embryos were incubated and electroporated as described above. After 24 h of reincubation (stage 21HH), the eggs were reopened and the embryo was covered with Teflon membrane (Fisher scientific, 13-298-83). The opening of the egg was sealed with low melting point agarose and covered with a layer of PBS. The internal GFP signal was imaged on Leica SP5 upright confocal microscope with a 40×/0.80 APO water dip-in lens about every 10 min over a period of 2 h. A z stack with steps of 1µm and total volume of about 90 µm was made for each time point. Images were processed with ImageJ.

### Computing

Information about basic 3D image processing for OPT can be found in supplementary methods Text S1.

#### Hardware

The simulations and parameter optimizations were performed on a Red Hat Enterprise Linux v5.3 cluster. In total 19 blades bl35p with 2 processors AMD Opteron Dual Core 2.4 GHz and 8 GB of RAM, 16 blades bl460c with 2 processors Xeon QuadCore E4540 and 18GB of RAM. All blades are running the Sun Grid Engine v6.2.

#### Navier–Stokes equation

The FEM solver used was Fastflo 3.1.2 in an adapted version to run in shell mode. An artificial compressibility method was use to derive the equation for pressure. From the continuity equation (5), we can assume that the pressure p satisfies a pseudo-transient state:
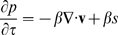
(10)where parameter *τ* is the pseudo-time and *β* is the artificial compressibility parameter [37]. Through a second-order Taylor expansion of the term 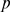 and substitution of the Navier–Stokes equation, we can arrive at a Poisson equation for pressure:
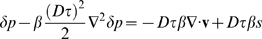
(11)where *Dτ* is the pseudo-time step. This is a convenient method to allow the ***s*** distribution to determine the pressure distribution, which in turn drives the resultant velocities (using equation 3). The third-order derivative of v can be neglected because we are dealing with very low values for 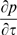. A zero-pressure boundary condition was chosen, and the equation was coded in *Fasttalk*, as in [56]. To solve for velocity with the Navier–Stokes equation, a slip flow boundary condition (zero normal velocity gradient) was chosen, apart from the flank boundary (junction between the limb bud and the embryo flank) which had the extra constraint that movement in the *x*-axis was not permitted. At each time step the vertex coordinates were updated by the local velocity values (a Lagrangian mesh approach) which allows the boundary conditions to stay in the correct position (on the edge of the limb bud shape) and also allows the **s** values to stay associated with the same tissue element over time.

#### Simulation with oriented cell movements

To implement a force-field with a similar set of orientations to the cell data, we combined two fields: the normal one generated by the measured proliferation rates (which is largely “outwards,” towards the ectoderm), and a new distally oriented field, which was defined as pointing unidirectionally along the PD axis, and whose magnitude increased in the distal direction.

#### RBF

Mapping the 30 Tc values onto the 3D tetrahedral mesh was a two-step process. First, 30 vertices within the mesh were chosen for their correspondence to the centres of the cell-counted regions. This gave us a sparse representation of proliferation for the limb. Second, an RBF was used to interpolate these 30 T_c_ values onto the remaining vertices of the *S_t0_* mesh. A RBF is a function approximation technique that can work in arbitrary dimensions. In this case a function is created using the 3D positions of the 30 selected vertices and corresponding T_c_ values as input. This function can then interpolate the T_c_ value for any 3D position. The software is coded in Matlab and is provided publicly on the Matlab Central website (www.mathworks.com) by Alex Chirokov [57].

#### Fitness function

The fitness function compared the two shapes, predicted pt_1_ and real shape t_1_ from the empirical dataset. It determines the quality of the match by measuring the distances of each surface vertex of pt_1_ to the closest triangle on the surface of t_1_. The distances are accumulated to a scalar value measurement of difference. We carried out various tests comparing a range of equal shapes and simple geometric shapes with different scalings and translations to test if the comparisons are reliable (unpublished data). The tests showed that this is the case for convex shapes like we are using in our optimization.

#### Cube mesh

To decrease the number of necessary optimizations we segmented the limb domain by creating a bounding box enclosing the limb mesh. This cube was subdivided into 9×9×9 cubes with a total of 729 vertices (Video S1). Each of the cube vertices is assigned a proliferation value which is linearly interpolated onto the tetrahedral limb mesh vertices. For cases when a cube vertex only belongs to cubes that don't contain tetrahedral limb mesh vertices, the cube vertex is deactivated and will not be used in the parameter optimization. This way the number of individual optimization steps was reduces from 729 to 525.

## Supporting Information

Figure S1 **Reproducibility of limb shapes.** Since the assessment of simulations depends on detailed shape information from two different specimens (for t0 and t1), it is important to confirm the general reproducibility of mouse limb bud shapes. (A) Six limb buds were chosen at stage E11.0. By extracting the outline of each limb (both from a dorsal view and a posterior view) we can overlay them to illustrate the minimal degree of shape variation. (C) The same data were obtained for limb buds 6 h older (E11.25). (B) By overlaying the two ages, we can confirm that the developmental shape change between the two stages is large by comparison with the variation within each group.
(3.00 MB TIF)

Figure S2 **Analysis of mesenchymal cell density.** (A) Graph of cell density against age of hindlimb bud. The two simulations in the paper correspond to the first and last third of this plot (E10.5–E10.75 and E11.0–E11.25). From this graph we could calculate that the density increases by 2.1% per hour for the younger time interval and 1.7% per hour for the older interval. (B) A limb bud of E11.25 was fluorescently stained for Sox9 expression—an early marker for the cartilage molecular prepattern. Two sections were imaged under two different fluorescent channels—the upper row shows only Sox9 expression (see Heckscher-Sorenson and Sharpe [58] for technical details). The inset panel illustrates where these sections lie on a photo of another whole-mount in situ for the same gene. (C–H) The lower row of square panels displays sub-regions from the two sections (positions shown as white squares in (B), in which only the fluorescent nuclear label is shown. In this way we were able to compare the cell density from Sox9-expressing regions (pre-skeletal) with the non Sox9 expressing mesenchyme. The cell counts show there is no obvious sign of mesenchymal condensation yet, despite the expression of Sox9.
(3.16 MB TIF)

Figure S3 **Effect of the Reynolds number on the simulations.** The resulting shapes of the simulation with five orders of magnitude difference in the Re number were compared. The measured PD elongation varies between 3% and 12%, whereas the real elongation is 43%.
(0.46 MB TIF)

Figure S4 **Volume-corrected simulations.** (A) The predicted shape change achieved with the cell-cycle times obtained by experimental data (as in Figure 4D). (B) Simulation in which the Tc values were scaled up to create the correct volume for the real t1 shape, proving that this correction does not improve the final predicted shape—the predicted and real t1 shapes still fail to match. (C–D) The same result for the E10.5 simulation.
(2.05 MB TIF)

Figure S5 **Mesenchymal movements in the absence of overlying ectoderm.** An E12 mouse limb bud was cultured in vitro for time-lapse imaging (see Boot et al. [59] for more technical details). After the initial adjustment phase, the limb bud shape changes were quite normal (although as usual for in vitro culture growth was slower than in utero). After about 12 h a tear appeared in the ventral ectoderm. This is highlighted at 13:30 with the asterisk. In the subsequent frames (every 1.5 h) the damaged edges of ectoderm can be seen slowly pulling away from each other (black arrowheads), revealing the mesenchyme underneath. Visual contrast in the mesenchymal tissue allowed us to track the positions of three points of tissue (white dots) over the remaining 11 h. These frames show that the living, growing mesenchyme does not spill out into the medium and instead continues to actively expand parallel to the PD axis, despite the absence of overlying ectoderm.
(0.45 MB TIF)

Figure S6 **Fitness landscape analysis.** The reliability of the parameter optimisation was assessed by starting from six different initial proliferation patterns. These patterns were not chosen randomly but specifically represent extreme alternative spatial distributions. The results show that, despite the dramatically different initial conditions, the algorithm always converges at a similar solution. All panels consist of a ventral view showing the initial proliferation pattern (top) and the optimized result (bottom). The following initial patterns were used: (A) AP-graded proliferation pattern with highest values on the anterior side, (B) a radial pattern with a central core of high proliferation, (C) high proliferation proximal and low values distal—this is the inverse of the optimized pattern and therefore requires a complete reversal of the pattern, (D) uniform zero proliferation, (E) a core of low proliferation in the center of the limb and high values nearer the ectoderm, and (F) a random spatial pattern.
(2.18 MB TIF)

Figure S7 **Oriented cell divisions in mouse limb buds.** To explore whether the bias in cell division orientation was conserved in the mouse we performed time-lapse imaging of mouse hindlimb buds in culture. Three mouse limb buds were labelled with bodipyceramide, cultured in vitro, and time-lapse imaged with confocal microscopy (see Supplementary Methods). This is an alternative measure of cell division orientation, compared to the chick analysis of relative chromatid positions during telophase. (A) Three frames from a time-lapse movie in which a dividing cell can be tracked. (B) The orientation bias of cell divisions from the three separate culture experiments. In all three cases the bias was seen approximately towards the closest ectoderm, as for the chick.
(0.38 MB TIF)

Figure S8 **Simulation with directional cell activities.** Since we have shown that isotropic growth alone is insufficient to explain limb bud shape changes, it is useful to perform a simulation which includes a directional tissue movement, to illustrate how the real growth patterns may appear. In this simulation we combined the measured distribution of isotropic growth (the s field, calculated from the Tc data and the measured changes in cell density), with a hypothetical distally oriented force-field f. The simple addition of this directional force is enough to transform the unsuccessful shape change (A) into a realistic velocity vector field (B) which produces a very accurate predicted shape (C). This reasonable fit to the empirical shape change was achieved just by optimizing the magnitude of the distal force. How the combination of possible directional cell activities (migration, cell intercalation, and cell division) combines to create a distally oriented resultant force is unclear but can hopefully be studied in more detail in the future.
(2.44 MB TIF)

Figure S9 **Flow chart of the parameter optimization process.** The flow chart demonstrates how the parameter optimization is implemented. A farmer thread splits the problem into smaller jobs that get distributed to a worker thread. When the worker is finished the farmer collects the result and integrates it into the current data set.
(0.60 MB TIF)

Text S1 **Supplementary methods.**
(0.04 MB DOC)

Video S1 **Discretization of space by the orthogonal cube mesh.** Although the limb bud itself is discretized into a tetrahedral mesh with 5,959 vertices, we employ a coarser orthogonal mesh to define the free parameters for optimizing the growth pattern. The full orthogonal mesh contains 729 vertices (9×9×9), but as some of the cube elements do not enclose any of the tetrahedral mesh, we can reduce the number of useful (optimized) vertices down to 525.
(5.98 MB AVI)

Video S2 **Optimisation of the 3D growth pattern.** Each frame of this movie shows one iteration of the optimization process, which gradually converges on a growth pattern producing a shape similar to the empirical t1. The distribution of colours changes as the growth pattern improves. The shape displayed in each frame is the resulting shape from a simulation with the current growth pattern (the transparent surface indicates the correct empirical t1 shape). Note this is not a movie of growth itself (in which the colours would not change, because the growth pattern is fixed for a given simulation).
(6.64 MB AVI)

## Abbreviations

AER: apical ectodermal ridge
AP: anterior-posterior
DV: dorsal-ventral
ECM: extra-cellular matrix
FEM: finite element model
OPT: optical projection tomography
PD: proximal-distal
*pS_t1_*: predicted result of the simulation
RBF: radial basis function
*S_t0_*: empirical measured limb shape of age E11.0
*S_t1_*: empirical measured limb shape of age E11.25
Tc: cell cycle time
ZPA: zone of polarizing activity

## References

[1] Wolpert L, Waddington C. H (1968). The French Flag problem: a contribution to the discussion on pattern development and regulation;.

[2] Wolpert L (1969). Positional information and the spatial pattern of cellular differentiation.. J Theor Biol.

[3] Gasseling M. T, Saunders J. W (1961). Effects of the apical ectodermal ridge on growth of the versene-stripped chick limb bud.. Dev Biol.

[4] Bell E, Saunders J. W, Zwilling E (1959). Limb development in the absence of ectodermal ridge.. Nature.

[5] Saunders J. W (2002). Is the progress zone model a victim of progress?. Cell.

[6] Saunders J. W (1972). Developmental control of three-dimensional polarity in the avian limb.. Ann N Y Acad Sci.

[7] Rubin L, Saunders J. W (1972). Ectodermal-mesodermal interactions in the growth of limb buds in the chick embryo: constancy and temporal limits of the ectodermal induction.. Dev Biol.

[8] Ede D. A, Law J. T (1969). Computer simulation of vertebrate limb morphogenesis.. Nature.

[9] Reiter R. S, Solursh M (1982). Mitogenic property of the apical ectodermal ridge.. Dev Biol.

[10] Niswander L, Martin G. R (1993). FGF-4 regulates expression of Evx-1 in the developing mouse limb.. Development.

[11] Wolpert L. H. A (1970). Cell division in the early growth and morphogenesis of the chick limb.. Nature.

[12] Dudley A, Ros M, Tabin C (2002). A re-examination of proximodistal patterning during vertebrate limb development.. Nature.

[13] Fernandez-Teran M. A, Hinchliffe J. R, Ros M. A (2006). Birth and death of cells in limb development: a mapping study.. Dev Dyn.

[14] Niswander L, Martin G. R (1992). Fgf-4 expression during gastrulation, myogenesis, limb and tooth development in the mouse.. Development.

[15] Prykhozhij S, Neumann C (2008). Distinct roles of Shh and Fgf signaling in regulating cell proliferation during zebrafish pectoral fin development.. BMC Developmental Biology.

[16] Xu J, Liu Z, Ornitz D. M (2000). Temporal and spatial gradients of Fgf8 and Fgf17 regulate proliferation and differentiation of midline cerebellar structures.. Development.

[17] Gong Y, Mo C, Fraser S. E (2004). Planar cell polarity signalling controls cell division orientation during zebrafish gastrulation.. Nature.

[18] Baena-López L, Baonza A, García-Bellido A (2005). The orientation of cell divisions determines the shape of Drosophila organs.. Curr Biol.

[19] Li S, Muneoka K (1999). Cell migration and chick limb development: chemotactic action of FGF-4 and the AER.. Dev Biol.

[20] Saunders J. W (1948). The proximo-distal sequence of origin of the parts of the chick wing and the role of the ectoderm.. J Exp Zool.

[21] Martin P, Lewis J (1986). Normal development of the skeleton in chick limb buds devoid of dorsal ectoderm.. Dev Biol.

[22] Dillon R, Othmer H. G (1999). A mathematical model for outgrowth and spatial patterning of the vertebrate limb bud.. J Theor Biol.

[23] Morishita Y, Iwasa Y (2008). Growth based morphogenesis of vertebrate limb bud.. Bull Math Biol.

[24] ten Berge D, Brugmann S. A, Helms J. A, Nusse R (2008). Wnt and FGF signals interact to coordinate growth with cell fate specification during limb development.. Development.

[25] Poplawski N. J, Swat M, Gens J. S, Glazier J. A (2007). Adhesion between cells, diffusion of growth factors, and elasticity of the AER produce the paddle shape of the chick limb.. Physica A.

[26] Sharpe J, Ahlgren U, Perry P, Hill B, Ross A (2002). Optical projection tomography as a tool for 3D microscopy and gene expression studies.. Science.

[27] Brooks R. A, Di Chiro G (1975). Theory of image reconstruction in computed tomography.. Radiology.

[28] Schöberl J (1997). NETGEN - an advancing front 2D/3D-mesh generator based on abstract rules.. ComputVisualSci.

[29] Dolbeare F, Gratzner H, Pallavicini M, Gray J (1983). Flow cytometric measurement of total DNA content and incorporated bromodeoxyuridine.. Proc Nat Acad Sci U S A.

[30] Martynoga B, Morrison H, Price D. J, Mason J. O (2005). Foxg1 is required for specification of ventral telencephalon and region-specific regulation of dorsal telencephalic precursor proliferation and apoptosis.. Dev Biol.

[31] Shibui S, Hoshino T, Vanderlaan M, Gray J (1989). Double labeling with iodo- and bromodeoxyuridine for cell kinetics studies.. Journal of Histochemistry and Cytochemistry.

[32] Murea C. M, Hentschel H. G (2007). A finite element method for growth in biological development.. Math Biosci Eng.

[33] Phillips H. M, Steinberg M. S, Lipton B. H (1977). Embryonic tissues as elasticoviscous liquids. II. Direct evidence for cell slippage in centrifuged aggregates.. Dev Biol.

[34] Berger S, Jou L (2000). Flows in stenotic vessels..

[35] Szczerba D, Székely G (2005). Computational model of flow–tissue interactions in intussusceptive angiogenesis.. Journal of Theoretical Biology.

[36] CSIRO (2006). FastFlo..

[37] Ferziger J, Peric M, Morton K (1999). Computational methods for fluid dynamics.

[38] Borkhvardt V. G (2000). [The growth and form development of the limb buds in vertebrate animals].. Ontogenez.

[39] Jaeger J, Surkova S, Blagov M, Janssens H, Kosman D (2004). Dynamic control of positional information in the early Drosophila embryo.. Nature.

[40] Reinitz J, Sharp D. H (1995). Mechanism of eve stripe formation.. Mech Dev.

[41] Ashyraliyev M, Jaeger J, Blom J. G (2008). Parameter estimation and determinability analysis applied to Drosophila gap gene circuits.. BMC Syst Biol.

[42] Kirkpatrick S, Gelatt C, Vecchi M (1983). Optimization by simulated annealing.. Science (New York, NY).

[43] Hooke R, Jeeves T (1961). “Direct search” solution of numerical and statistical problems.. JACM.

[44] Ridley A, Schwartz M, Burridge K, Firtel R, Ginsberg M (2003). Cell migration: integrating signals from front to back.. Science.

[45] Holmes L. B, Trelstad R. L (1977). Patterns of cell polarity in the developing mouse limb.. Dev Biol.

[46] Ede D, Wilby O (1981). Golgi orientation and cell behaviour in the developing pattern of chondrogenic condensations in chick limb-bud mesenchyme.. The Histochemical Journal.

[47] Holmes L. B, Trelstad R. L (1980). Cell polarity in precartilage mouse limb mesenchyme cells.. Dev Biol.

[48] Shih J, Keller R (1992). Cell motility driving mediolateral intercalation in explants of Xenopus laevis.. Development.

[49] Lewis J (1975). Fate maps and the pattern of cell division: a calculation for the chick wing-bud.. Development.

[50] Summerbell D, Wolpert L (1972). Cell density and cell division in the early morphogenesis of the chick wing.. Nature: New Biology.

[51] Qian D, Jones C, Rzadzinska A, Mark S, Zhang X (2007). Wnt5a functions in planar cell polarity regulation in mice.. Developmental Biology.

[52] Yamaguchi T, Bradley A, McMahon A, Jones S (1999). A Wnt5a pathway underlies outgrowth of multiple structures in the vertebrate embryo.. Development.

[53] Nowakowski R. S, Lewin S. B, Miller M. W (1989). Bromodeoxyuridine immunohistochemical determination of the lengths of the cell cycle and the DNA-synthetic phase for an anatomically defined population.. J Neurocytol.

[54] Suzuki T, Ogura T (2008). Congenic method in the chick limb buds by electroporation.. Development, Growth & Differentiation.

[55] Kulesa P. M, Fraser S. E (2000). In ovo time-lapse analysis of chick hindbrain neural crest cell migration shows cell interactions during migration to the branchial arches.. Development.

[56] Z Zhu A. N. S (1995). Computation of swirling turbulent diffusion flames with a finite-element method.. Proc 12th Australasian Fluid Mechanics Conference.

[57] Chirokov A (2006). Scattered data interpolation and approximation using radial base functions..

[58] Hecksher-Sorensen J, Sharpe J (2001). 3D confocal reconstruction of gene expression in mouse.. Mech Dev.

[59] Boot M. J, Westerberg C. H, Sanz-Ezquerro J, Cotterell J, Schweitzer R (2008). In vitro whole-organ imaging: 4D quantification of growing mouse limb buds.. Nat Methods.

