# Supplementary figures and images for: The Role of Spatially Controlled Cell Proliferation in Limb Bud Morphogenesis
Source: PLoS Biol. 2010 Jul 13;8(7):e1000420. doi: 10.1371/journal.pbio.1000420 (PMC2903592; doi:10.1371/journal.pbio.1000420)

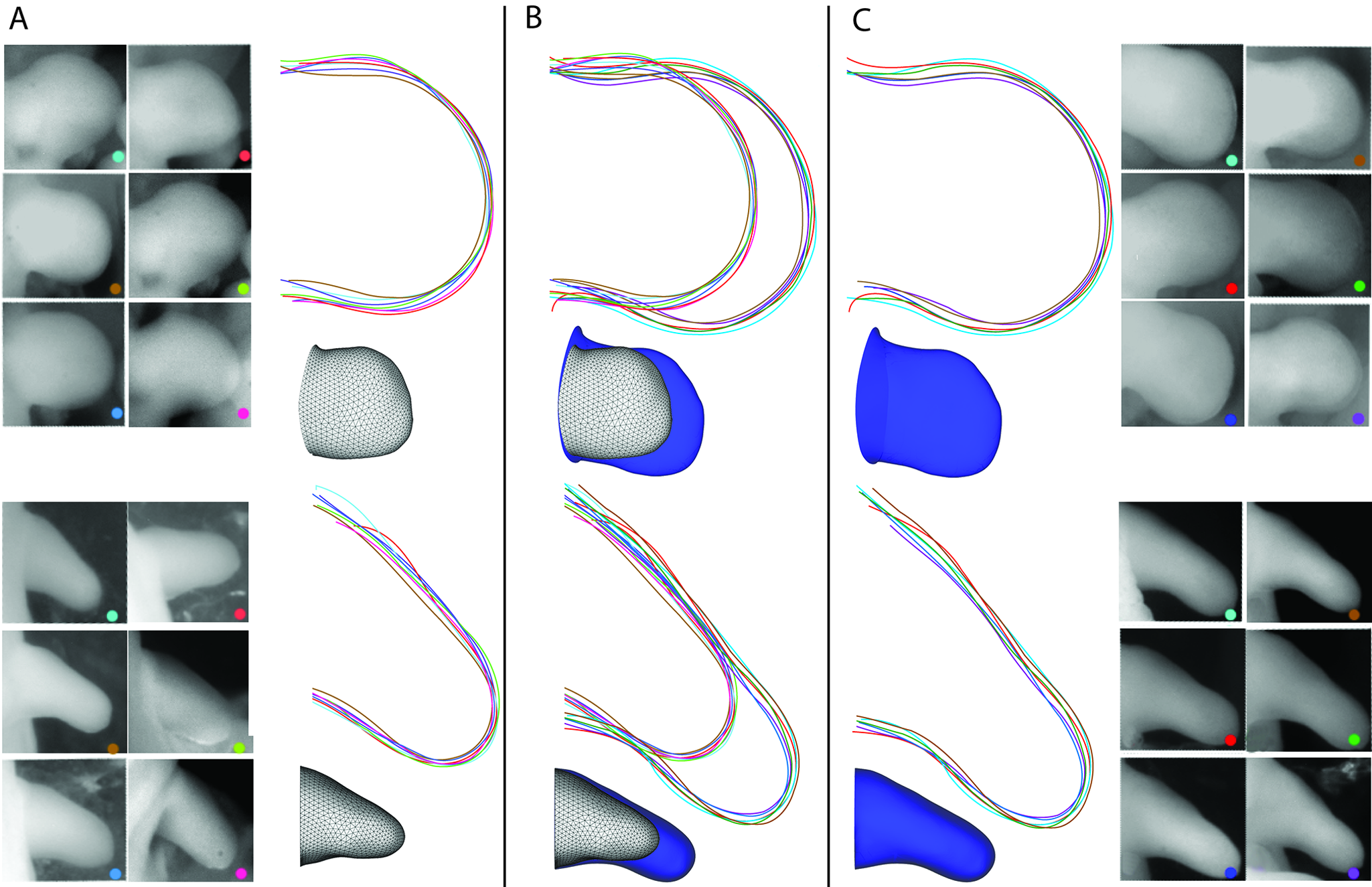

Supplement: Figure S1 — Reproducibility of limb shapes. Since the assessment of simulations depends on detailed shape information from two different specimens (for t0 and t1), it is important to confirm the general reproducibility of mouse limb bud shapes. (A) Six limb buds were chosen at stage E11.0. By extracting the outline of each limb (both from a dorsal view and a posterior view) we can overlay them to illustrate the minimal degree of shape variation. (C) The same data were obtained for limb buds 6 h older (E11.25). (B) By overlaying the two ages, we can confirm that the developmental shape change between the two stages is large by comparison with the variation within each group. (3.00 MB TIF) [file pbio.1000420.s001.tif]

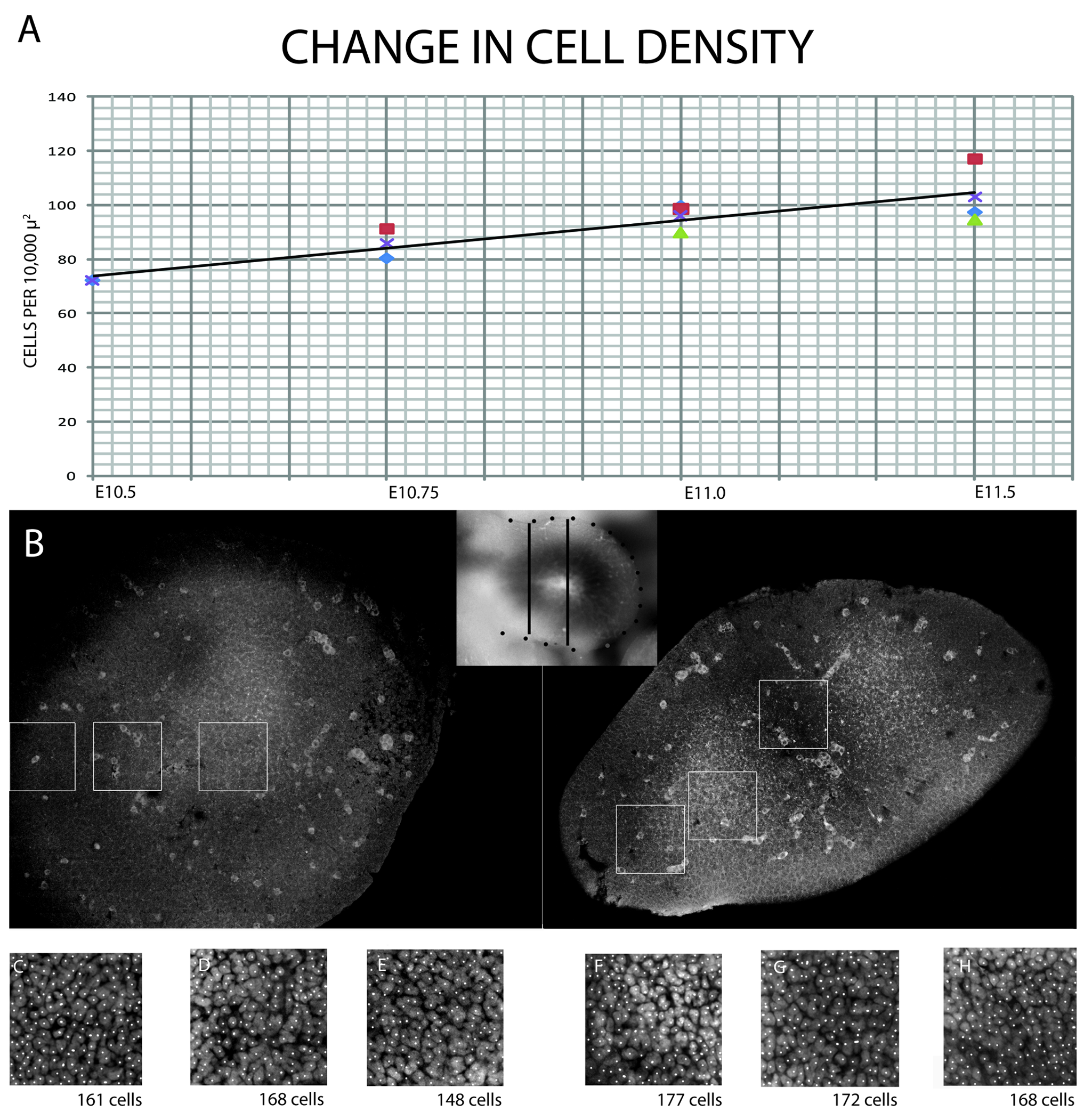

Supplement: Figure S2 — Analysis of mesenchymal cell density. (A) Graph of cell density against age of hindlimb bud. The two simulations in the paper correspond to the first and last third of this plot (E10.5–E10.75 and E11.0–E11.25). From this graph we could calculate that the density increases by 2.1% per hour for the younger time interval and 1.7% per hour for the older interval. (B) A limb bud of E11.25 was fluorescently stained for Sox9 expression—an early marker for the cartilage molecular prepattern. Two sections were imaged under two different fluorescent channels—the upper row shows only Sox9 expression (see Heckscher-Sorenson and Sharpe [58] for technical details). The inset panel illustrates where these sections lie on a photo of another whole-mount in situ for the same gene. (C–H) The lower row of square panels displays sub-regions from the two sections (positions shown as white squares in (B), in which only the fluorescent nuclear label is shown. In this way we were able to compare the cell density from Sox9-expressing regions (pre-skeletal) with the non Sox9 expressing mesenchyme. The cell counts show there is no obvious sign of mesenchymal condensation yet, despite the expression of Sox9. (3.16 MB TIF) [file pbio.1000420.s002.tif]

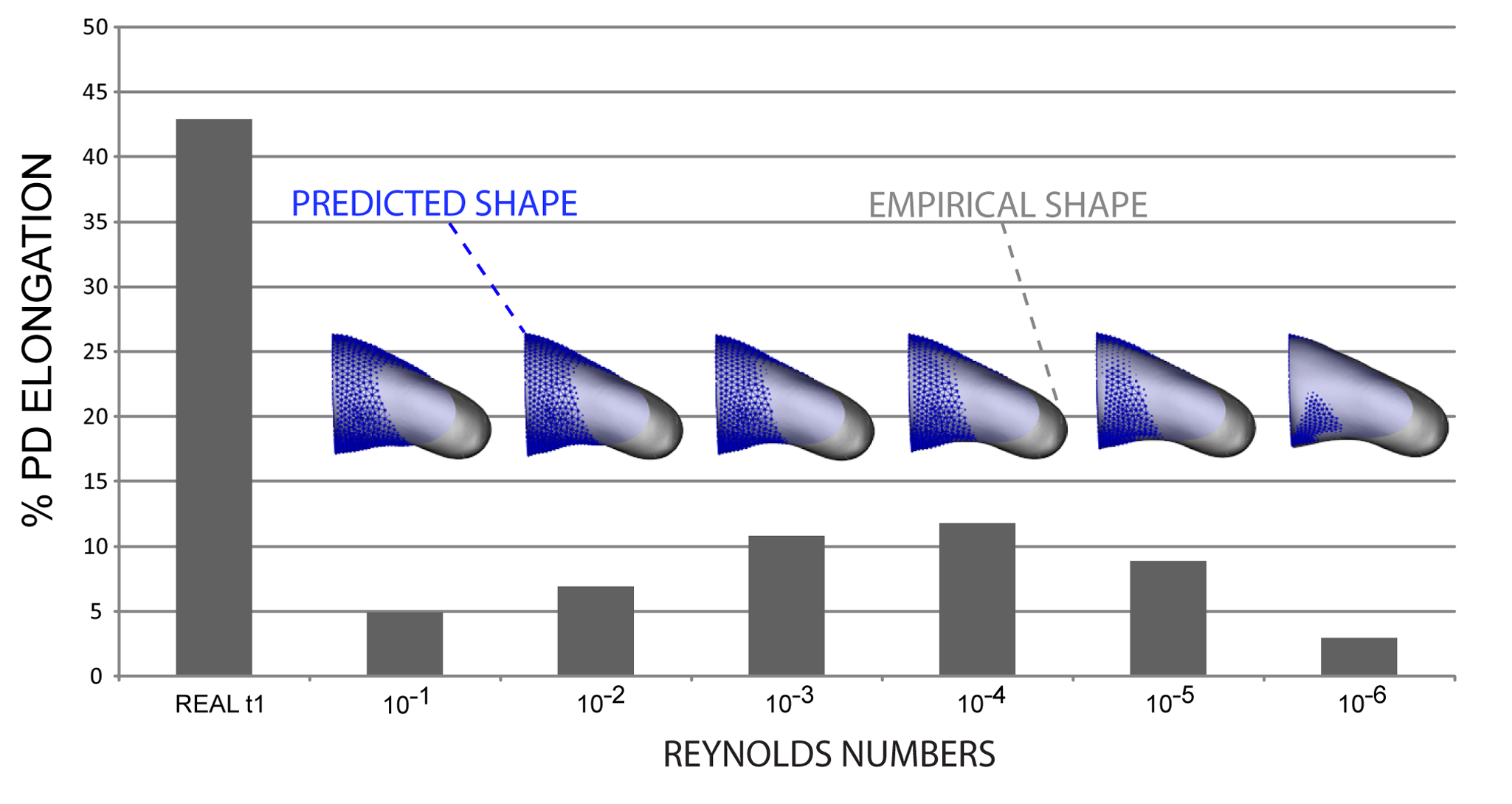

Supplement: Figure S3 — Effect of the Reynolds number on the simulations. The resulting shapes of the simulation with five orders of magnitude difference in the Re number were compared. The measured PD elongation varies between 3% and 12%, whereas the real elongation is 43%. (0.46 MB TIF) [file pbio.1000420.s003.tif]

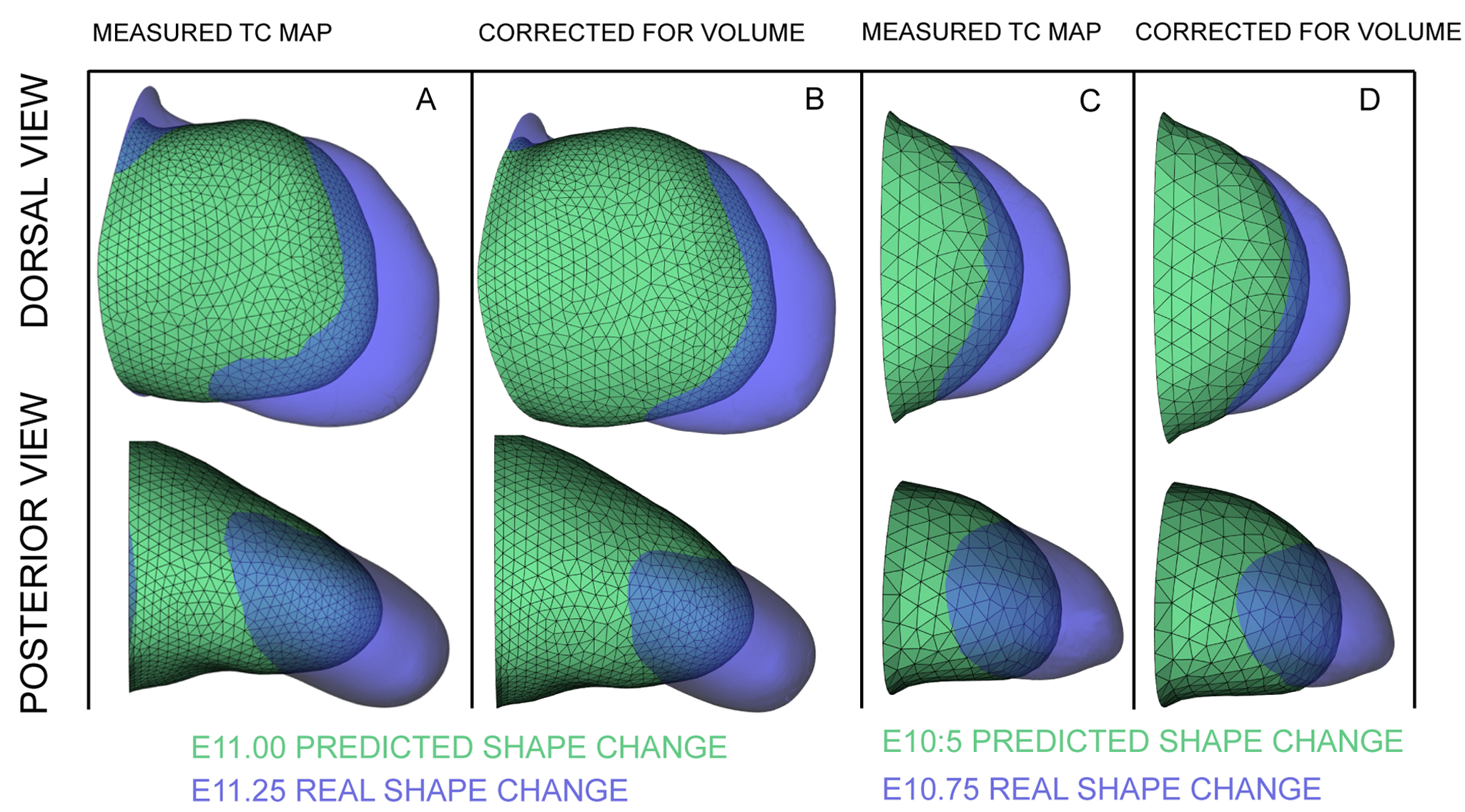

Supplement: Figure S4 — Volume-corrected simulations. (A) The predicted shape change achieved with the cell-cycle times obtained by experimental data (as in Figure 4D). (B) Simulation in which the Tc values were scaled up to create the correct volume for the real t1 shape, proving that this correction does not improve the final predicted shape—the predicted and real t1 shapes still fail to match. (C–D) The same result for the E10.5 simulation. (2.05 MB TIF) [file pbio.1000420.s004.tif]

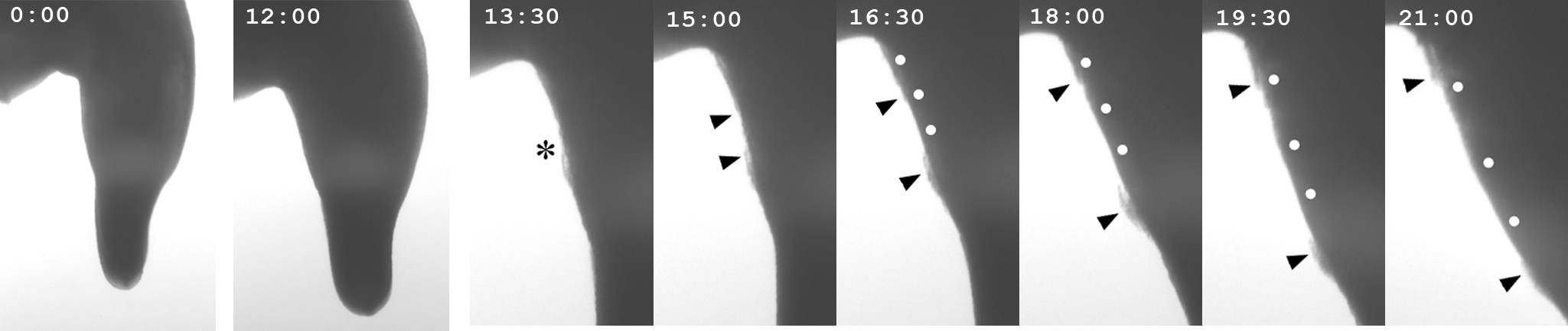

Supplement: Figure S5 — Mesenchymal movements in the absence of overlying ectoderm. An E12 mouse limb bud was cultured in vitro for time-lapse imaging (see Boot et al. [59] for more technical details). After the initial adjustment phase, the limb bud shape changes were quite normal (although as usual for in vitro culture growth was slower than in utero). After about 12 h a tear appeared in the ventral ectoderm. This is highlighted at 13:30 with the asterisk. In the subsequent frames (every 1.5 h) the damaged edges of ectoderm can be seen slowly pulling away from each other (black arrowheads), revealing the mesenchyme underneath. Visual contrast in the mesenchymal tissue allowed us to track the positions of three points of tissue (white dots) over the remaining 11 h. These frames show that the living, growing mesenchyme does not spill out into the medium and instead continues to actively expand parallel to the PD axis, despite the absence of overlying ectoderm. (0.45 MB TIF) [file pbio.1000420.s005.tif]

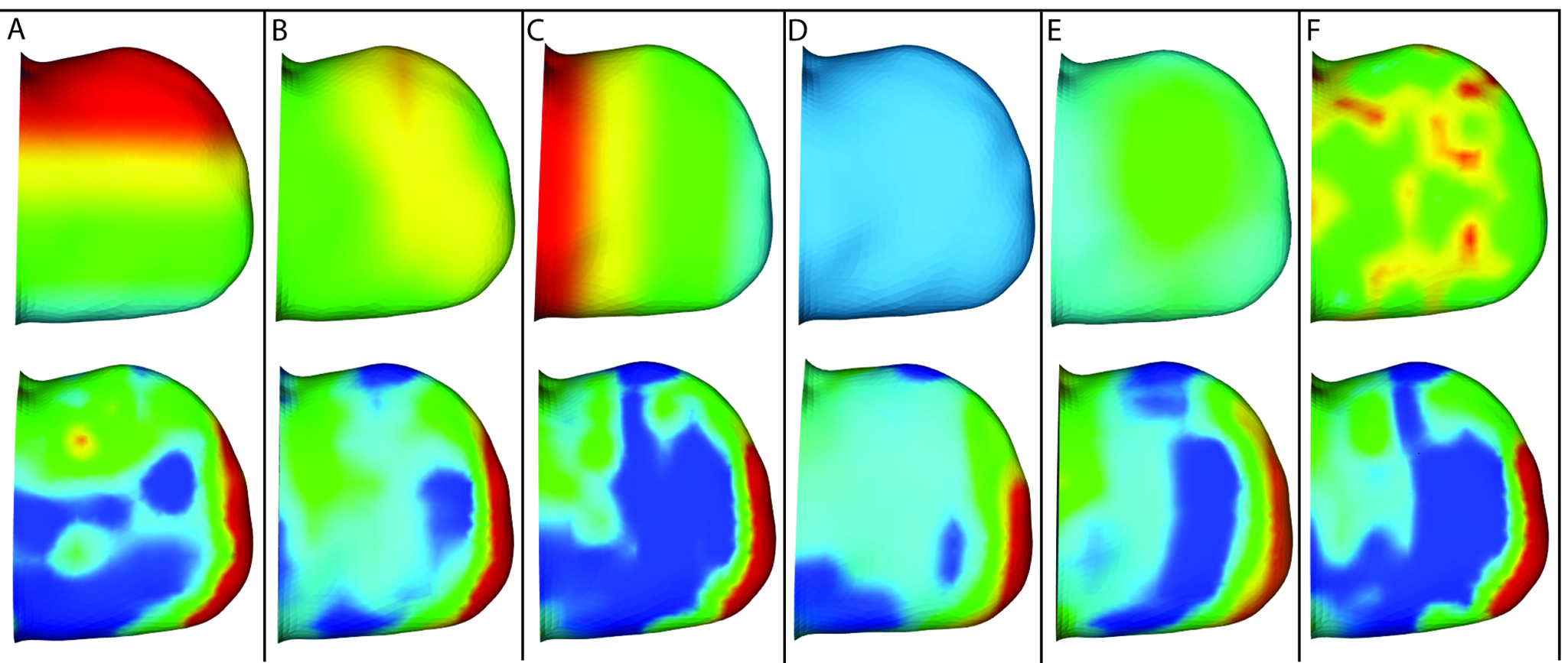

Supplement: Figure S6 — Fitness landscape analysis. The reliability of the parameter optimisation was assessed by starting from six different initial proliferation patterns. These patterns were not chosen randomly but specifically represent extreme alternative spatial distributions. The results show that, despite the dramatically different initial conditions, the algorithm always converges at a similar solution. All panels consist of a ventral view showing the initial proliferation pattern (top) and the optimized result (bottom). The following initial patterns were used: (A) AP-graded proliferation pattern with highest values on the anterior side, (B) a radial pattern with a central core of high proliferation, (C) high proliferation proximal and low values distal—this is the inverse of the optimized pattern and therefore requires a complete reversal of the pattern, (D) uniform zero proliferation, (E) a core of low proliferation in the center of the limb and high values nearer the ectoderm, and (F) a random spatial pattern. (2.18 MB TIF) [file pbio.1000420.s006.tif]

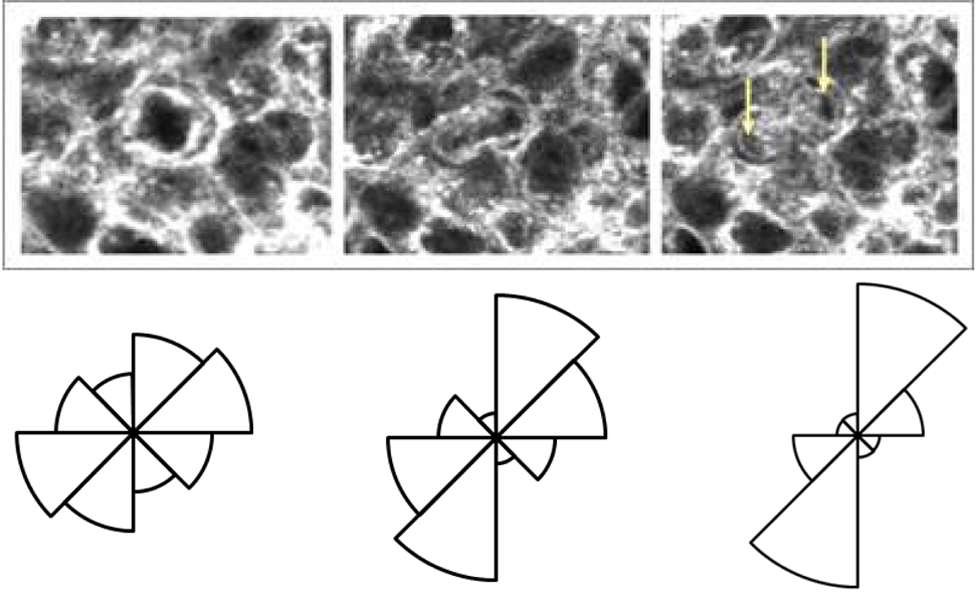

Supplement: Figure S7 — Oriented cell divisions in mouse limb buds. To explore whether the bias in cell division orientation was conserved in the mouse we performed time-lapse imaging of mouse hindlimb buds in culture. Three mouse limb buds were labelled with bodipyceramide, cultured in vitro, and time-lapse imaged with confocal microscopy (see Supplementary Methods). This is an alternative measure of cell division orientation, compared to the chick analysis of relative chromatid positions during telophase. (A) Three frames from a time-lapse movie in which a dividing cell can be tracked. (B) The orientation bias of cell divisions from the three separate culture experiments. In all three cases the bias was seen approximately towards the closest ectoderm, as for the chick. (0.38 MB TIF) [file pbio.1000420.s007.tif]

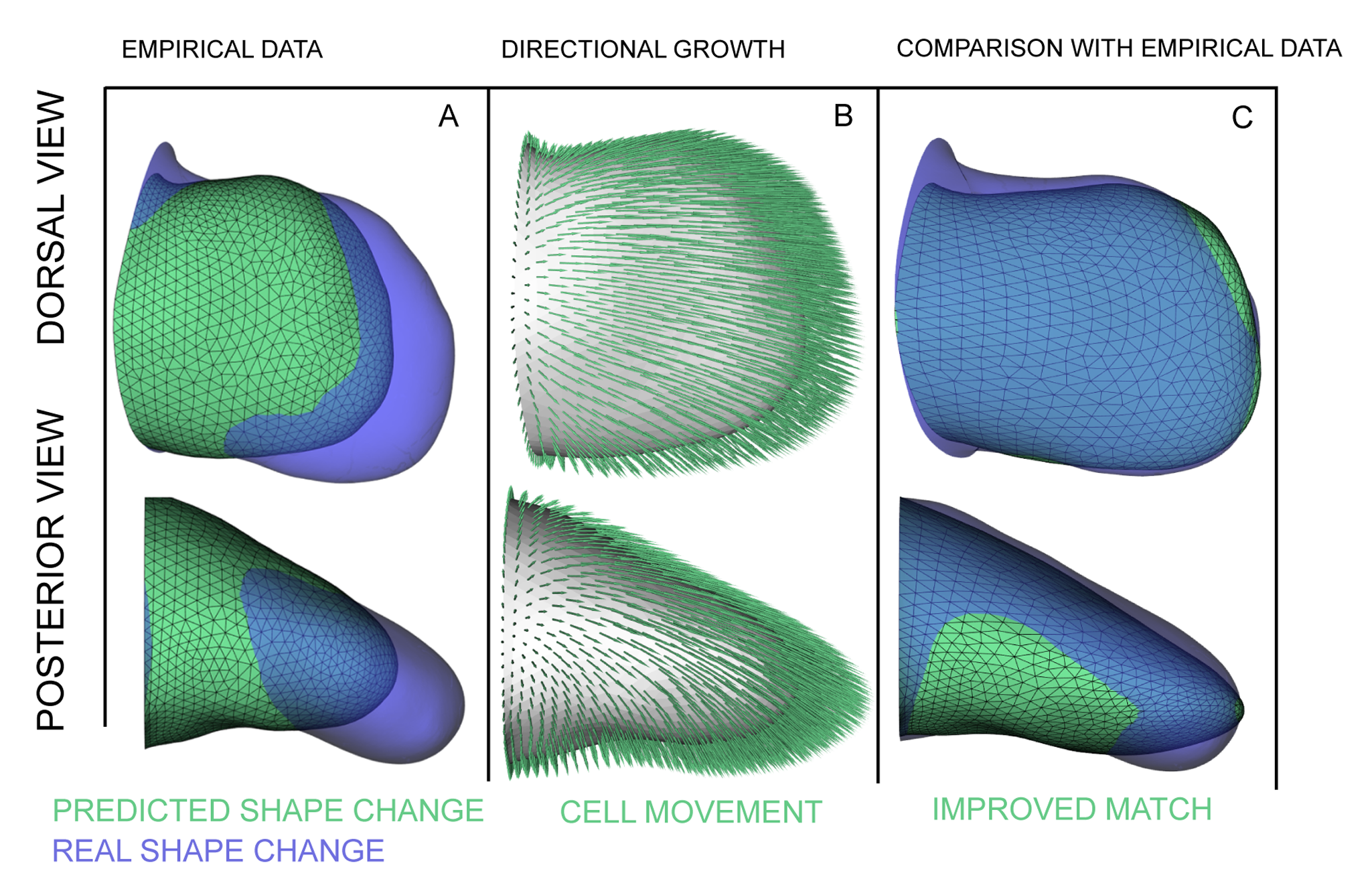

Supplement: Figure S8 — Simulation with directional cell activities. Since we have shown that isotropic growth alone is insufficient to explain limb bud shape changes, it is useful to perform a simulation which includes a directional tissue movement, to illustrate how the real growth patterns may appear. In this simulation we combined the measured distribution of isotropic growth (the s field, calculated from the Tc data and the measured changes in cell density), with a hypothetical distally oriented force-field f. The simple addition of this directional force is enough to transform the unsuccessful shape change (A) into a realistic velocity vector field (B) which produces a very accurate predicted shape (C). This reasonable fit to the empirical shape change was achieved just by optimizing the magnitude of the distal force. How the combination of possible directional cell activities (migration, cell intercalation, and cell division) combines to create a distally oriented resultant force is unclear but can hopefully be studied in more detail in the future. (2.44 MB TIF) [file pbio.1000420.s008.tif]

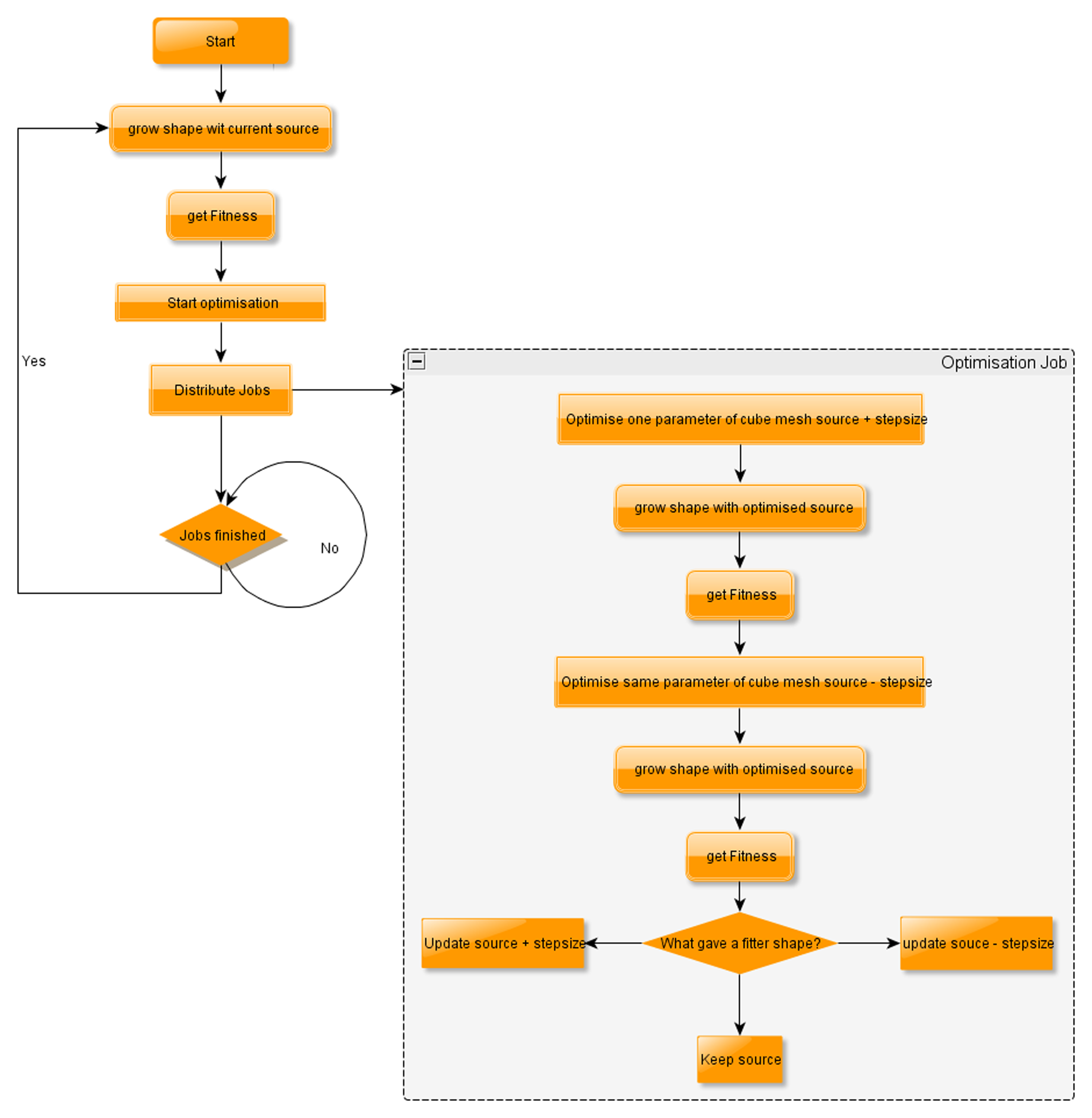

Supplement: Figure S9 — Flow chart of the parameter optimization process. The flow chart demonstrates how the parameter optimization is implemented. A farmer thread splits the problem into smaller jobs that get distributed to a worker thread. When the worker is finished the farmer collects the result and integrates it into the current data set. (0.60 MB TIF) [file pbio.1000420.s009.tif]
