# Supplementary material for: The Role of Spatially Controlled Cell Proliferation in Limb Bud Morphogenesis
Source: PLoS Biol. 2010 Jul 13;8(7):e1000420. doi: 10.1371/journal.pbio.1000420 (PMC2903592; doi:10.1371/journal.pbio.1000420)
Supplement: Text S1 — Supplementary methods. (0.04 MB DOC) [file pbio.1000420.s010.doc]

# **The role of spatially-controlled cell proliferation in limb bud morphogenesis.**

**Supplementary information**

Contents

[Supplementary Methods 2](#__RefHeading___Toc260240022)

[Creating a Voxel data set 2](#__RefHeading___Toc260240023)

[Creating an un-refined iso surface 2](#__RefHeading___Toc260240024)

[Bodipy Ceramide Staining and imaging 3](#__RefHeading___Toc260240025)

[References: 3](#__RefHeading___Toc260240026)

## Supplementary Methods

## Creating a Voxel data set

The t_0_ and t_1_ meshes are both derived from OPT scans of two different whole embryos. The first step in generating a mesh is to create a voxel reconstruction for each scan. A reconstruction is 3D voxel data set, where each voxel is a cube with a scalar intensity value. The voxels are arranged in to 3D cube that reflect the 3D data in a scan. A reconstruction is created by using a back-projection algorithm to calculate the intensity of a given voxel based on the images within an OPT scan, as described in [1].

## Creating an un-refined iso surface

Initial iso-surface generation is handled by VTK (Visualisation Toolkit (www.vtk.org)) an open soure, cross platform software library for 3D computer graphics and visualisation and has interfaces to Tcl/Tk, Java, and Python. Iso-surface generation involves applying a series of geometric filters to the 3D voxel data that involves a number of computational processing steps: voxel data smoothing, scaling, thresholding, surface mesh smoothing. As we are only interested in the hind limbs, these are manually cropped out of the voxel data set before the surface generation steps. The cropping is made to leave plenty of room around the limb and where the limb joins the body to give sufficient space to close the limb surface at a later step.

Smoothing is the first step, done by 2D Gaussian blurring. For this a radius is specified in terms of pixels. The “Marching Cubes” algorithm is then used to generate a 2D surface that represents a boundary in the 3D voxel data which is formed based on a given threshold. A triangle surface smoothing filter is then applied which relaxes the vertices of the triangles to produce a smoother surface by making the triangles have better shapes and having the vertices more evenly distributed. Finally the surface is scaled by the pixel size so that the surface is to scale (1 voxel = 1 micron).

For the t_0_ shape the values are: 6 pixel radius Gaussian smoothing, iso-surface threshold 19, triangle smoothing relaxation factor 0.3, number of iterations 10, pixel size 11.61. The same values or the t_1_ shape and a pixel size of 12.35.

The values for the smoothing and thresholding were found through trial and error by experimenting with different combinations of values visually trading off overall limb smoothness without loss of shape.

Aligning and closing

Before the surfaces can be tetrahedralized the limbs need to be closed as this is a requirement of mesh creation. In addition, the two limbs occupy different spaces spatially so a rigid body transform is applied to each surface so that they overlay well. A clipping plane is then used to separate the limbs from the body and the clip provides information on how close the limb surfaces.

Mesh Generation

Mesh generation is the process of taking a 3D surface and filling the volume with 3D elements. In our case we choose tetrahedral (pyramids) for the FEM (fastflo). The closed surfaces are passed to NETGEN (www.hpfem.jku.at/netgen) , an open-sources 3D surface and volume mesh generator, to be further optimised, during surface re-generation and mesh generation. The surface goes through a second surface optimisation step as it forms the basis for the resulting mesh and having a very high quality triangulation is important for meshing. The main options used in mesh generation are: *mesh size* and *chart distance* as these were loose controls on the number of elements generated by the program. Also the surface and resulting mesh went through a number of optimisation steps in order to improve the quality.

The values for the t_0_ mesh were: mesh size=0.1, chart distance=4.5, surface optimisation steps= 10, volume optimisation steps=5. The t_1_ surface is also generated using the same value but it did not require the full mesh generation, as the surface is sufficient for fitness calculations.

## Bodipy Ceramide Staining and imaging

Basic culture medium consisted of Dulbeccos modified medium with 10% Foetal Calf Serum, 1% Penicillin-Streptomycin. Wildtype E10.5 and E11.5 CD1 mice embryos were harvested and the trunk regions encompassing the hind limbs immediately dissected in basic culture medium. The trunk regions were stained in 100μl of staining solution made up of basic culture medium with bodipyceramide- FL (Molecular Probes) at a concentration of 100μM, at 37°C and 5% CO2. Left hind limbs were then time-lapse imaged in an FCS2 chamber (Bioptechs) with basic culture medium at 37°C. Embryos were imaged using a Zeiss LSM510 confocal microscope using a laser excitation wavelength of 488nm and a 63x oil immersion objective. The orientation of each observed cell division was measured using the angle tool in the LSM Image Browser software.

# References:

1. Sharpe J, Ahlgren U, Perry P, Hill B, Ross A, et al. (2002) Optical projection tomography as a tool for 3D microscopy and gene expression studies. Science 296: 541-545.
